# Simulations of Two-Photon Absorption Spectra of Fluorescent Dyes: The Impact of Non-Condon Effects

**DOI:** 10.1021/acs.jctc.4c01545

**Published:** 2025-03-26

**Authors:** Rudraditya Sarkar, Carmelo Naim, Karan Ahmadzadeh, Robert Zaleśny, Denis Jacquemin, Josep M. Luis

**Affiliations:** † Institute of Computational Chemistry and Catalysis and Department of Chemistry, University of Girona, Campus de Montilivi, 17003 Girona, Catalonia, Spain; ‡ Department of Chemistry, School of Science, Gandhi Institute of Technology and Management (GITAM), Hyderabad 502329, India; § Nantes Université, CNRS, CEISAM UMR 6230, F-44000 Nantes, France; ∥ Hefei National Research Center for Physical Sciences at the Microscale, University of Science and Technology of China, Hefei, Anhui 230026, China; ⊥ Faculty of Chemistry, Wrocław University of Science and Technology, Wyb. Wyspiańskiego 27, PL-50370 Wrocław, Poland; # Institut Universitaire de France (IUF), F-75005 Paris, France

## Abstract

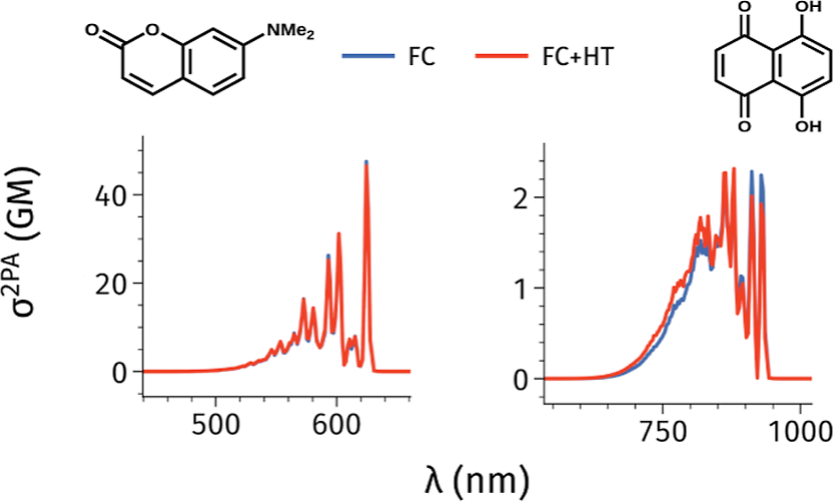

Computer simulations play a pivotal role in interpreting experimental two-photon absorption (2PA) spectra. One of the key aspects of the simulation of these spectra is to take into account the vibrational fine structure of the bands in electronic spectra. This is typically done by employing Franck–Condon (FC) term and low-order terms in the Herzberg–Teller (HT) expansion. In this work, we present a systematic study of first-order HT effects on the vibronic structure of π → π* electronic bands in 2PA spectra of 13 common fluorophores. We begin by evaluating the performance of several density functional approximations (DFAs) against the second-order coupled cluster singles and doubles model (CC2) for reproducing two-photon transition moments and their first- and second-order derivatives with respect to normal modes of vibration on a set of six donor–acceptor molecules. Our findings reveal that most DFAs produce inaccurate values for these derivatives, with the exception of the LC-BLYP functionals with range–separation parameters of 0.33 and 0.47. Although these functionals underestimate the HT contribution to the 2PA total intensities of the π → π* electronic bands, they offer a reasonable qualitative reproduction of the HT vibrational fine structure of the reference spectra. We further explore HT effects on fluorescent chromophores, finding that HT contributions are secondary to FC effects, leading to small shifts of the wavelengths peaks, and minimal changes in the intensities. Additionally, the adiabatic Hessian, vertical Hessian, and vertical gradient vibronic models are assessed. The general agreement among these models confirms that the harmonic approximation is suitable for studying the selected fluorophores.

## 1 Introduction

Multiphoton absorption phenomena, with two-photon absorption (2PA) at their forefront, are currently in the limelight of both experimental and computational studies. The main rationale explaining this interest in 2PA processes is their potential in key technological applications, e.g., three-dimensional microfabrication,^1^ optical data storage,^2^ and bioimaging.^3,4^ Besides, 2PA can also be invaluable in spectral investigations of atoms and molecules. Indeed, as highlighted by Neusser and Schlag, the 2PA measurement setup might allow obtaining high-resolution spectra below the Doppler width.^5^ 2PA spectroscopy is also advantageous to tackle questions related to symmetry,^6,7^ in particular, the characterization of states that are dark with usual one-photon absorption (1PA) spectroscopies. These dark states might be strictly forbidden due to selection rules (e.g., due to the centrosymmetry of the molecule, as defined by the well-known Laporte’s rule) or simply too weak to be observed with 1PA approaches. An interesting illustration is given by the S_0_ → S_2_ transition in Reichardt’s dye which presents a strong signal in the 2PA spectra but a very weak one in its 1PA counterpart.^8^ Because of its potential for characterizing electronic structure, 2PA spectroscopy was used during the 70s and 80s of the past century to probe the electronic structure of conjugated polyenes and other important chromophores.^7,9^

This large panel of applications explains why one can find a plethora of studies aiming at establishing structure–property relationships for two-photon absorbers.^10−14^ Unsurprisingly, experimental interpretations of 2PA observations have been supported by theoretical simulations since the 1970s.^15−17^ The high interpretive power of computational models remains exploited today to understand the nonlinear absorption activity.^18−20^ This has been accompanied by the constant extension of the palette of methods allowing determining purely electronic two-photon transition strengths.^21−28^ However, in order to model the band shapes in electronic 2PA spectra of molecules, one also needs to account for molecular vibrations.

Luo, Ågren, and collaborators have pionneered this field by performing the first ab initio calculations of the vibrationally resolved 2PA spectra.^29−32^ During the last two decades several groups also contributed to these efforts.^33−37^ These works demonstrated that computer simulations of vibrationally resolved 1PA and 2PA spectra are invaluable for interpreting the observed spectroscopic features. Indeed, absorption bands corresponding to the same electronic state might yield quite different shapes in the two types of spectra, as illustrated by the experimental data recorded for the chromophores of fluorescent proteins.^38^ These differences were attributed to non-Condon effects,^39,40^ and such effects might be potentially important in other fluorophores.^41^ Moreover, accurate modeling of 2PA spectra may reduce the need for complex experiments that often rely on highly sensitive detection systems and powerful laser sources.^42^

The primary objective of the present work is to provide a systematic evaluation of the importance of non-Condon effects in 2PA spectra across a significant range of small fluorescent probes. We propose using DFT for this analysis, as it has been observed to provide qualitatively valuable results for purely electronic 2PA with a good balance between accuracy and computational cost.^20,43−50^ However, the ability of DFT to capture vibronic effects has been largely overlooked. Consequently, we assess here the influence of the selected density functional approximation (DFA) on key parameters, notably, the second-order transition moments and their derivatives, comparing the data to wave function methods to appraise both their reliability and accuracy.

## 2 Methods

### 2.1 First-Principle Approaches

The macroscopic expression of the 2PA is generally quantified as the cross section of simultaneous absorption of two isoenergetic photons, that can be expressed as^51^
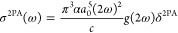
1where α, *a*_0_, *c*, ω, *g*(2ω) and δ^2PA^ respectively represent the fine structure constant, the Bohr radius, the speed of light, the frequency of the incident photon, the broadening function and the rotationally averaged two-photon transition strength. In this study we assume Gaussian line-shape function (with arbitrary width) for all molecules for comparative assessment (see the Results and Discussion for details), and the given broadening values refer to half width at half-maximum (HWHM). The two-photon absorption cross section, σ^2PA^(2ω), is measurable experimentally, and it is generally expressed in Göppert-Mayer units (GM) in honor of Maria Göppert-Mayer who first theorized 2PA processes,^52^ where 1 GM = 10^–50^ cm^4^·s·photon^–1^. δ^2PA^ can be evaluated through electronic structure methods, and it can be expressed in terms of second-order transition moments (*S*_ab_) as^53^
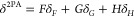
2where δ_*F*_, δ_*G*_, and δ_*H*_ are respectively given by
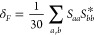
3
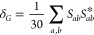
4
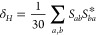
5and *F*, *G*, *H* are polarization variables. In what follows we assume one source of linearly polarized photons (*F* = *G* = *H* = 2). We underline that δ_*F*_, δ_*G*_, and δ_*H*_ involve products of left and right second-order transition moments in the case of non-Hermitian theories.^23^ Note that the components of *S*_ab_ tensor, involving sum over states, can be evaluated using purely electronic or vibronic wave functions. In this work, we analyze the effect of non-Condon effects by using the Herzberg–Teller expansion of *S*_ab_ tensor components with respect to vibrational normal modes (*Q*)
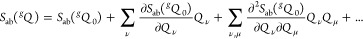
6where ^*g*^*Q*_0_ is the initial state equilibrium geometry, *S*_ab_(^*g*^*Q*_0_) indicates the *S*_ab_ tensor at the ^*g*^*Q*_0_ geometry, whereas  and  indicate the first- and second-order derivatives with respect to the normal mode (*Q*_*v*_). Detailed theoretical developments can be found in refs (33–35 and 37). The linear and higher-order terms with respect to *Q* account for non-Condon effects (e.g., the second-order term introduces electrical anharmonicity). In this study we simulate the vibronic spectra retaining only up to the linear term in eq 6. Moreover, the performance of various electronic-structure theories is compared for second-order normal-mode derivatives of selected *S*_ab_ tensor elements. We obtained such parameters using three electronic-structure codes. The GAUSSIAN 16 program^54^ was used to perform geometry optimizations and vibrational structure calculations, while the GAMESS US^26,55^ and VeloxChem^56^ programs were used to compute the elements of the second-order transition moment tensor. Normal-mode derivatives were determined numerically using an in-house code and the numerical stability was controlled with the aid of the Rutishauser–Romberg approach.^57,58^ To that end, we used mesh of 10 displacements per coordinate (±2^*k*^Δ, *k* = 0–4; in the case of displacements in Cartesian coordinates Δ was set to 0.005 Bohr). The analysis of Romberg triangles is performed as outlined by Medved et al.^58^

To perform a comparative assessment of the *S*_ab_ derivatives, we selected as references the results obtained with the second-order coupled-cluster CC2 model^59^ with the resolution-of-identity approximation (RI-CC2)^23^ as implemented in the TURBOMOLE 7.6 program.^60,61^ In the TD-DFT framework, the palette of tested DFAs encompasses the semilocal generalized gradient approximation (GGA) representatives (PBE^62^ and BLYP^63,64^), the global hybrid GGA (B3LYP^65^ and PBE0^66,67^), the range-separated GGA (LC-BLYP^68^), and the global hybrid *meta-*GGA (MN15^69^) exchange–correlation functionals. Note that the response theory calculations of *meta-*GGAs were performed with the gauge-variant formulation of the kinetic energy τ, however, the differences with respect to gauge-invariant results are not expected to be significant given the nature of the investigated electronic transitions.^70,71^ For LC-BLYP we used the software default values, i.e., μ = 0.33 (GAMESS US) and μ = 0.47 (GAUSSIAN 16), and we additionally checked other values of the range–separation parameter μ for molecules from ref (48). We tested several different values of μ as well as its optimal tuning based on Janak’s theorem.^48^ Based on the statistical analysis reported in Table S1 of the Supporting Information we include μ = 0.10 in our set of DFAs. To simplify the notation, we append the decimal values of μ directly in the names of the functionals in the following, i.e., LC-BLYP10, LC-BLYP33, and LC-BLYP47.

**Scheme 1:**
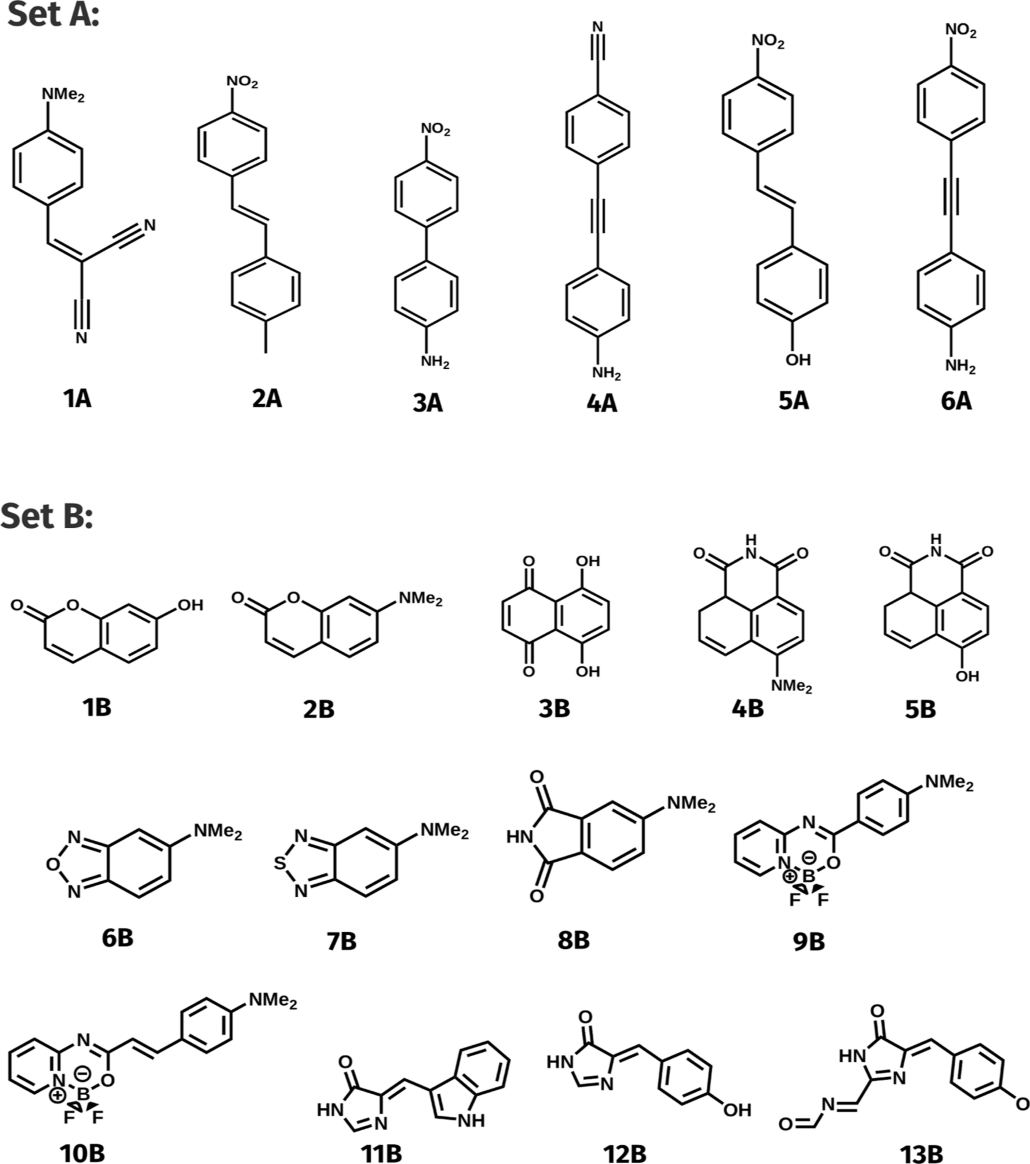
Push-Pull Molecules Used for Analyzing the Performances of DFA (Set A), and Common Fluorophores (Set B) Considered in This Study

The simulations of vibronic 2PA spectra were performed at the TD-DFT level within the mechanical harmonic approximation,^30,72^ using the FCClasses program.^73,74^ Various vibronic models are available, each differing in the representation of the potential energy surfaces (PES) of the ground state (GS) and the target excited state (ES). Among these models, the adiabatic Hessian (AH), vertical Hessian (VH), and vertical gradient (VG) approaches are the most commonly employed. The AH method is the most computationally demanding, as it approximates the PES by considering the minima and harmonic vibrations of both the GS and ES equilibrium geometries. In contrast, the vertical models approximate the ES minimum based on the ES Hessian (VH) or gradient (VG) at the GS equilibrium geometry.^72^ In this study, we use the VG model to assess the performance of the DFAs and evaluate the importance of non-Condon effects in simulating the 2PA spectra of the first set of molecules, Set A in Scheme 1; moreover, we conduct a qualitative comparison of the three models in the 2PA simulations of the second set of molecules, i.e., Set B in Scheme 1. While these differences do not provide direct guidance on which method to select—since this decision often relies either on convergence of the different models or on direct comparisons with experimental data—they offer valuable insights into the suitability of the harmonic approximation. Indeed would the harmonic approximation be exact for the studied PESs, there would be no differences between AH and VH methods. Hence, when anharmonic effects become important, the three methods can yield substantially different spectral shapes. For calculations using the VH model, all frequencies were considered real (positive) at the excited state PES.

Following the terms present in the HT expansion of the *S*_ab_ moments, we analyze three distinct types of vibronic 2PA spectra: (i) Franck–Condon spectra (FC, including the *S*_ab_(^*g*^*Q*_0_) term only, i.e., neglecting any dependence on *Q*_ν_), (ii) HT spectra (FC + HT, both *S*_ab_ and linear terms with respect to *Q*_ν_ are included), (iii) pure HT spectra (HT, only linear term with respect to *Q*_ν_ is included, FC contributions are discarded). Since for the investigated states, the purely electronic transitions are allowed (δ^2PA^ ≠ 0), the pure HT spectra have no physical meaning. The latter simulations were performed to pinpoint the effect of the normal-mode derivatives of the second-order transition moment on the vibronic band shapes. All vibronic calculations were performed for temperature 0 K using the time-dependent (TD) formulation and rely on Cartesian coordinates. FC + HT spectra were generated using the so-called HTi approach of FCClasses (i.e., derivatives were evaluated around the initial state geometry), while pure HT contributions have been computed by setting *S*_ab_(^*g*^*Q*_0_) = 0 for all tensor components. The Pople’s 6–31+G(d) atomic basis set^75^ is employed for all the computations of geometries, frequencies, and 2PA transitions, unless otherwise stated. The vibronic spectra simulations using AH, VG, and VH were performed using the same set of 2PA properties (*S*, d*S*/d*Q*) evaluated at the equilibrium geometry of the initial state.

### 2.2 Molecular Sets

For assessing various DFAs, we selected a series of π-conjugated push–pull molecules constituting Set A in Scheme 1. These molecules exhibit moderate 2PA responses, reaching up to dozens of GM at absorption band maximum. As a reference, we note that the topologically similar donor−π–acceptor dye 4-dimethylamino-4′-nitrostilbene shows 2PA cross-section as large as 116 GM in CHCl_3_ solution.^76^ It should be highlighted that all transitions have a π → π* character. Such transitions can typically be quite accurately described by single-determinant methods, including the RI-CC2 approach used as reference herein. Indeed, it has been shown by some of us that it reliably reproduces vertical δ^2PA^ when dealing with such states.^77^ The B3LYP/cc-pVTZ optimized geometries of the molecules of set **A** can be found in ref (48).

To evaluate non-Condon effects, we investigated a series of substituted fluorophores shown in Set B in Scheme 1, exhibiting meaningful σ^FC^ values (greater than 1 GM) for one of their lowest ESs. This set includes eight common organic dyes, two dyes carrying a BF_2_ group that have demonstrated large experimental 2PA,^78^ and three chromophores found in fluorescent proteins. The corresponding geometries optimized at the LC-BLYP33/6-31+G* are reported in the Supporting Information. As mentioned above, all considered excitations correspond to π → π* transitions to the first excited state in all molecules. The computed vibrationally resolved 1PA of these molecules, along with their experimental spectra in low-polarity solvents (when available), and corresponding references can be found in Table S8 and Figure S3 in the Supporting Information. These spectra do not show significant HT effects as applying the FC + HT method does not significantly alter the 1PA spectra obtained using the FC model. Furthermore, this approximation qualitatively captures the shape of the experimental spectra as illustrated in Figure S3 of the Supporting Information.

## 3 Results and Discussion

The accuracy of the computed vibronic 2PA spectra depends on the following key factors: (i) the distribution of vibronic transitions; (ii) the electronic two-photon transition strength at the equilibrium geometry and, presumably, (iii) non-Condon effects (involving geometric derivatives of second-order transition moments). All three aspects are considered below. First, we evaluate the performance of DFAs in predicting transition moments and their derivatives with respect to normal modes on molecules of set A. Subsequently, we assess the magnitude of HT effects and the impact of the choice of the vibronic model on molecules of set B.

### 3.1 Benchmark of Density Functional Approximations

**Figure 1:**
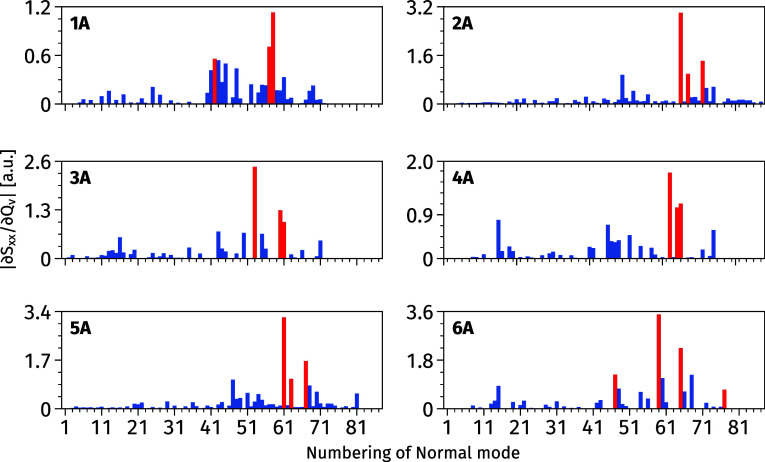
First derivative of the second-order transition moment *S*_xx_ with respect to normal modes for molecules **1A**-**6A** calculated at the LC-BLYP47/6-31+G(d) level of theory. Red vertical lines correspond to the subset of normal modes selected for the in-depth analyses—see text for details.

The numerical evaluation of geometrical derivatives of second-order transition moment is a computationally expensive task, especially at the RI-CC2 level, explaining why we focus on a subset of normal modes. The selection is performed both with respect to the number of modes and the number of components of the *S*_ab_ tensor. Our goal is to select no more than 5 vibrational normal modes and one tensor component for each molecule from set **A**. The selection procedure of the subset of normal modes was performed as follows. First, the complete set of first derivatives with respect to normal modes  was obtained for molecules **1A**–**6A** with the LC-BLYP47 functional. The statistical analysis in terms of average, median, and maximum value (with corresponding mode number ν) for all tensor components  was performed. The summary presented in Table 1 indicates a predominant contribution from the longitudinal *S*_*xx*_ tensor element. Additionally, the significant difference between the median and the average values for all studied molecules suggests a nonstandard distribution. The median-mean discrepancy and the large difference between the maximum and average values indeed indicate that most of the normal modes possess low  values. This is also seen in Figure 1 where the  values are plotted for each normal mode ν.

**Table 1:** Statistical Analysis of Normal Mode Derivatives of Second-Order Transition Moments (in a.u.) for Molecules **1A**–**6A**^a^

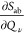	average	median	max (ν)	average	median	max (ν)	average	median	max (ν)
	**1A**			**2A**			**3A**		
*xx*	0.114	0.023	1.130 (58)	0.161	0.075	3.003 (65)	0.163	0.026	2.450 (53)
*yy*	0.007	0.002	0.062 (60)	0.004	0.002	0.041 (65)	0.004	0.002	0.037 (53)
*zz*	0.002	0.001	0.038 (63)	0.002	0.001	0.014 (65)	0.003	0.001	0.025 (55)
*xy*	0.024	0.010	0.325 (60)	0.015	0.007	0.090 (74)	0.013	0.003	0.124 (62)
*xz*	0.008	0.000	0.070 (65)	0.006	0.004	0.032 (65)	0.007	0.002	0.043 (58)
*yz*	0.002	0.000	0.043 (64)	0.002	0.001	0.009 (65)	0.002	0.001	0.013 (34)
	**4A**			**5A**			**6A**		
*xx*	0.136	0.006	1.764 (62)	0.200	0.070	3.193 (61)	0.217	0.003	3.489 (59)
*yy*	0.003	0.000	0.029 (54)	0.005	0.003	0.041 (61)	0.003	0.000	0.033 (59)
*zz*	0.001	0.000	0.012 (58)	0.002	0.001	0.018 (61)	0.001	0.000	0.013 (66)
*xy*	0.012	0.000	0.133 (59)	0.018	0.008	0.096 (61)	0.013	0.000	0.117 (67)
*xz*	0.003	0.000	0.025 (24)	0.006	0.004	0.035 (61)	0.002	0.000	0.024 (24)
*yz*	0.001	0.000	0.016 (37)	0.002	0.001	0.013 (61)	0.001	0.000	0.012 (39)

a All results were obtained at the LC-BLYP47/6-31+G(d) level of theory.

The selection of the dominant longitudinal component *S*_xx_ and its derivatives with respect to three (molecules **1**–**5**) or four (molecule **6**) vibrational modes for assessing DFAs was performed examining the data presented in Table 1 and Figure 1. In more detail, we selected the first derivatives of *S*_xx_ with respect to the following vibrational modes: ν_42_, ν_57_, and ν_58_ (**1**); ν_65_, ν_67_, and ν_71_ (**2**); ν_53_, ν_60_, and ν_61_ (**3**); ν_62_, ν_64_, and ν_65_ (**4**); ν_61_, ν_63_, and ν_67_ (**5**); ν_47_, ν_59_, ν_65_, and ν_77_ (**6**). In the latter case, we included the values of  with respect to four vibrational normal modes because the first derivatives with respect to the two in-plane scissoring bending vibrational modes (ν_47_ and ν_65_) present high and similar values. The selected vibrational modes are highlighted in red in Figure 1, while the atomic displacements corresponding to the considered vibrational modes can be found in Figure S1 and Table S2 in the Supporting Information.

The *S*_xx_ values calculated at the equilibrium geometry of the selected molecules employing several DFAs as well as RI-CC2 with the cc-pVDZ atomic basis set can be found in Table S3 in the Supporting Information. The corresponding statistical analysis is given in Table S4 in the Supporting Information and the ordering of the DFAs follows the ranking provided by the mean absolute percentage error (MAPE). It turns out that the *meta*-GGA global hybrid MN15 is superior to other DFAs with a MAPE of 15%, while both B3LYP and PBE0 provide less satisfactory results with respective MAPEs of 31% and 25%. Interestingly, conventional RSH functionals, LC-BLYP33 and LC-BLYP47, perform worse with respective MAPEs of 41% and 48%. In contrast, LC-BLYP10 is more satisfying with a MAPE of 26%, consequently outperforming B3LYP. Finally, as expected, a poor performance is found for the two evaluated semilocal GGA functionals, PBE and BLYP, with MAPE of 52%.

The values of the 19  obtained with different DFAs and RI-CC2 are provided in Table S5 in the Supporting Information, while the corresponding statistical analysis is presented in Table 2. Here, we used normal modes determined at LC-BLYP47/6-31+G(d,p) level of theory for computing derivatives at all other levels of theory, i.e., numerical derivatives were evaluated based on displacements along normal modes obtained from the above-mentioned method. As expected the relative errors of all DFAs with respect to RI-CC2 are much larger when considering derivatives rather than *S*_xx_. In contrast to what was observed for *S*_xx_, MN15 underperforms with respect to LC-BLYP33 and LC-BLYP47 in all indicators but the mean absolute error (MAE). In particular, the MAPE indicates that the most adequate functionals are LC-BLYP33 (38%) and LC-BLYP47 (40%), followed by MN15 (53%). PBE0 and B3LYP are the next functionals with large MAPEs of 74% and 83%, respectively, while LC-BLYP10 delivers a MAPE of 90%. Again, GGA functionals are unsatisfactory with very large MAPEs of ca. 130%. In short, LC-BLYP33 is the “best” functional (lowest MAPE), and therefore it will be used to account for the HT effects in the simulations of the vibronic 2PA spectra of molecules from set **B**.

**Table 2:** Statistical Analysis of  (a.u.) Performed for 19 Vibrational Modes of Molecules **1A**–**6A**^a^

DFA	MAPE [%]	MAE [a.u.]	SDE [a.u.]	RMSE [a.u.]	MAX AE [a.u.]
LC-BLYP33	38	0.91	1.22	1.2	3.82
LC-BLYP47	40	0.94	1.26	1.23	3.97
MN15	53	0.86	1.31	1.3	3.86
PBE0	74	1.36	2.01	1.97	5.71
B3LYP	83	1.52	2.22	2.17	6.2
LC-BLYP10	90	1.76	2.39	2.34	6.18
PBE	129	2.44	3.28	3.2	7.49
BLYP	131	2.47	3.3	3.23	7.51

a The errors are calculated with respect to the RI-CC2 results. The data are ordered according to increasing MAPE. The cc-pVDZ atomic basis set was used in all calculations.

We also computed the second derivatives of *S*_xx_ with respect to vibrational normal modes . These derivatives are necessary to account for the electrical anharmonicity in the simulation of vibronic 2PA spectra. This analysis was done for a subset of modes where numerical stability was satisfactory, i.e., numerical errors in  were less than 4% ( we underline that this rather large value corresponds to one outlier, the numerical errors are less than 0.1% for the majority of cases). In more detail, we report the data related to 6 modes of molecules **1A** and **4A** in Table S6 in the Supporting Information, and the relative statistical analysis in Table 3.

**Table 3:** Statistical Analysis of  (a.u.) Performed for 6 Vibrational Modes of Molecule **1A** and **4A**^a^

DFA	MAPE [%]	MAE [a.u.]	SDE [a.u.]	RMSE [a.u.]	MAX AE [a.u.]
LC-BLYP47	38	0.013	0.017	0.017	0.034
LC-BLYP33	48	0.015	0.020	0.019	0.043
MN15	102	0.029	0.040	0.037	0.062
PBE0	153	0.047	0.067	0.063	0.112
LC-BLYP10	155	0.046	0.064	0.059	0.091
B3LYP	171	0.053	0.077	0.073	0.138
PBE	215	0.060	0.081	0.076	0.118
BLYP	219	0.062	0.083	0.078	0.128

a The errors are calculated with respect to RI-CC2 results. The data are ordered according to increasing MAPE. The cc-pVDZ atomic basis set was employed in all calculations.

**Figure 2:**
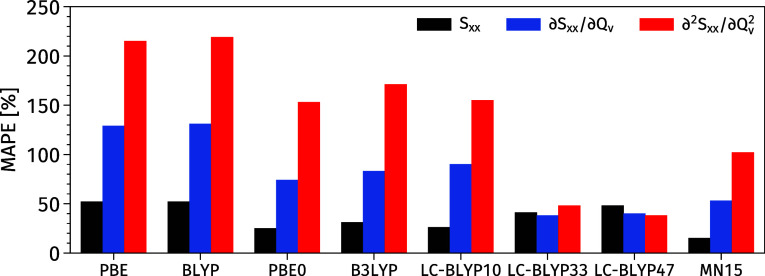
MAPE of *S*_xx_, , and  for molecules **1A**–**6A** using the RI-CC2 results as benchmarks. The first-order derivatives are performed on the selected 24 vibrational modes of molecules **1A**–**6A**, while the second-order derivatives on the 6 modes of molecules **1A** and **4A** (see the text for the list of modes included). The cc-pVDZ atomic basis set is employed.

The data given in Table 3 indicate that the performance of the two standard LC-BLYP variants is very good in the case of second derivatives. Indeed the errors (MAE, SDE, RMSE, and MAPE) obtained with LC-BLYP47 and LC-BLYP33 are almost half of their MN15 counterparts, which ranks third in the list. In more detail, the first two functionals have a MAPE value of 38% and 48% respectively, while the MN15 MAPE is 102%. The other DFAs deliver unsatisfactory estimations of the second derivatives with MAPE larger than 100%. LC-BLYP10, PBE0, and B3LYP deliver similar inaccuracies (MAPE between 155% and 171%) whereas PBE (215%) and BLYP (219%) are particularly poor. A summary of MAPE for all properties examined in this Section can be found in Figure 2.

**Figure 3:**
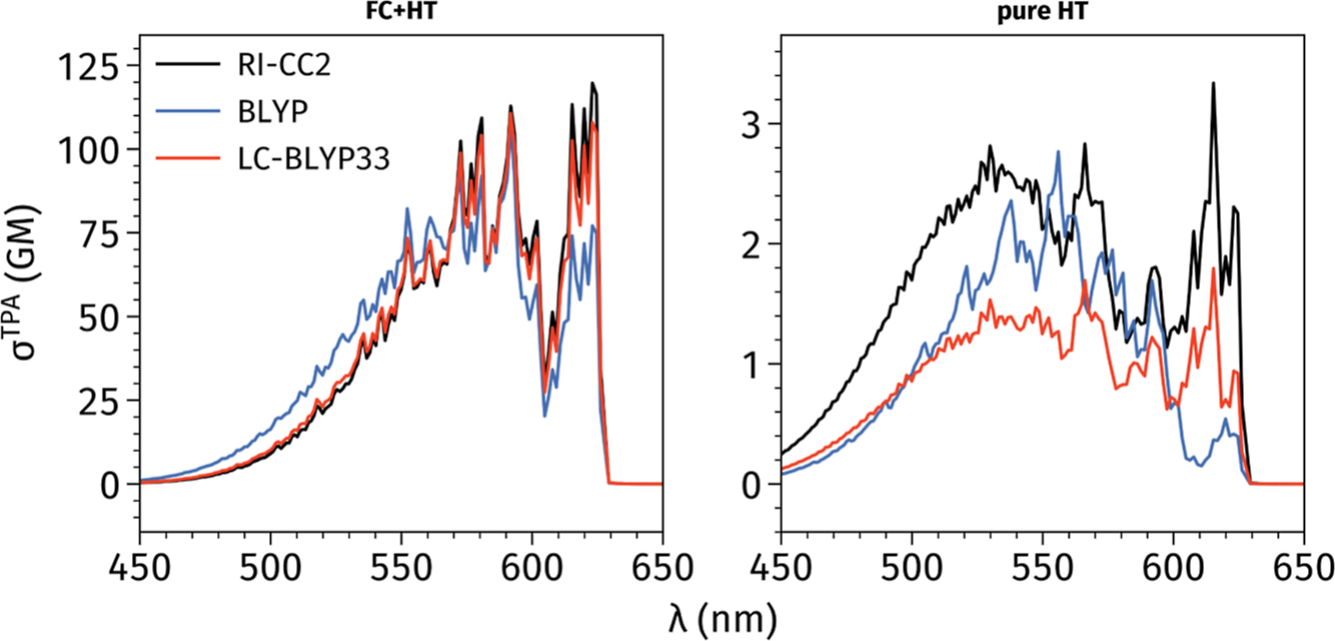
Vibronic FC + HT (left) and pure HT (right) 2PA spectra for molecule **6A**.

Let us now assess the impact of  derivatives, as given by different DFAs, on the vibronic 2PA spectra of molecules **1A**-**6A**. A comparison of the band shapes simulated using  for the selected normal modes obtained from the most accurate LC-BLYP33, least accurate BLYP, and reference RI-CC2 levels of theory is presented in Figure 3 for molecule **6A** (see also Figure S2 in the Supporting Information). The rationale behind the inclusion of the BLYP functional in this part of the study is to demonstrate how significant statistical errors determined for individual components affect the simulated 2PA band shapes. We recall that we determined all the  values using LC-BLYP47/6-31+G(d) only, while these derivatives for selected subset of vibrational normal modes were also computed at the RI-CC2, LC-BLYP33, and BLYP levels. Hence, to illustrate how the choice of DFA affects the 2PA band topologies, we use the *S*_ab_ and  values determined at the LC-BLYP47/6-31+G(d) level, replacing the derivatives for the selected modes with those computed at the RI-CC2, LC-BLYP33, and BLYP levels. In other words, for a given DFA (or RI-CC2) the Franck–Condon contribution and derivatives for 3*N*-9 (3*N*-10 for **6A**) modes are determined at LC-BLYP47/6-31+G(d) level, while the remaining derivatives for 3 (4 for **6A**) key modes are determined at DFA(RI-CC2)/cc-pVDZ level.

Hence, it comes as no surprise that Figure 3 and Figure S2 in the Supporting Information show similar peak positions for all three methods, i.e., all schemes restore the FC contribution as predicted by LC-BLYP47/6-31+G(d) which are predominant for these systems. However, analyzing pure HT vibronic spectra (with no FC contributions) one notices that the Herzberg–Teller spectra for **6A** reveal that the relative peak intensities might strongly differ when using RI-CC2 (or LC-BLYP33) or BLYP. These substantial changes are due to the differences in the magnitudes of  derivatives for four key normal modes only. It is clear that HT contributions, which affect the relative intensities of the peaks, crucially depend on the level of theory. We stress that LC-BLYP33 predicts a very similar intensity pattern as RI-CC2 but the intensities of individual peaks are much smaller. In contrast, the shape of the pure HT spectra predicted by BLYP strongly differs from its RI-CC2 counterpart. This outcome can be easily explained using the data of Table S5 of the Supporting Information, i.e., one finds that derivative values computed using LC-BLYP33 are roughly half their RI-CC2 counterparts. Finally, we underline that some DFAs deliver wrong derivative signs compared to RI-CC2 (see Table S5 of the Supporting Information).

### 3.2 Importance of HT Contributions in Common Dyes

**Figure 4:**
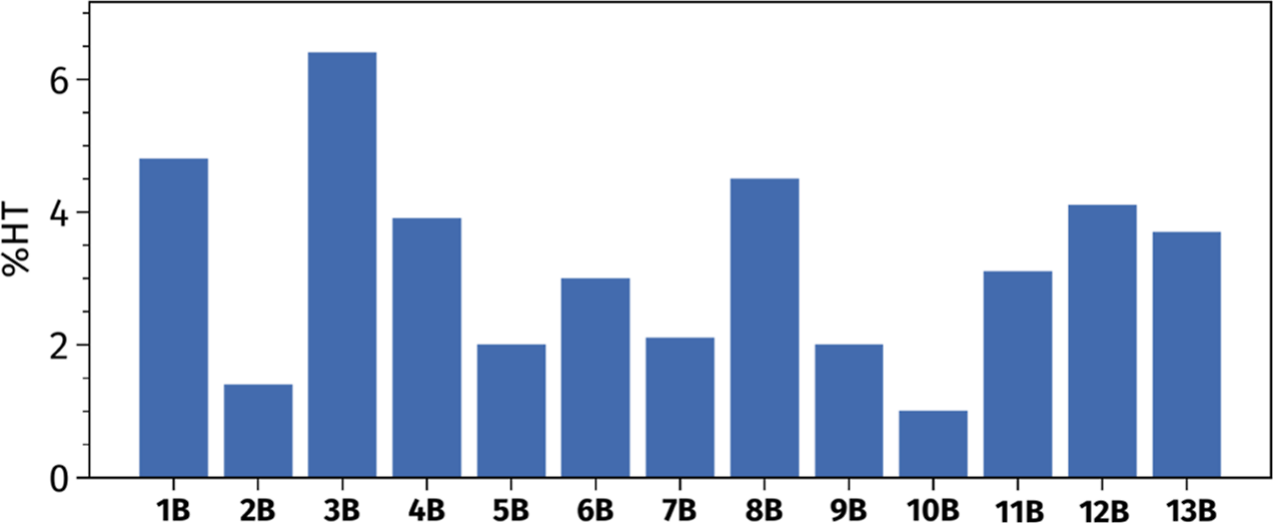
Percentage contributions of HT effects (% HT) on the total intensity of the studied π → π* 2PA electronic transitions for for **1B**–**13B**. See eqs 7 and 8.

Based on the above results, the LC-BLYP33 functional was used to compute the geometries and vibronic 2PA spectra of the **1B**–**13B** molecules. To estimate the impact of HT effects on the total intensity of a 2PA electronic band, δ^2PA^, we can separate the FC and HT effects by using the expression for total intensity at 0 K^30^
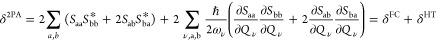
7where δ^FC^ includes the FC contributions to the total 2PA intensity, while δ^HT^ provides the HT contribution. In Figure 4 we report the percentage contribution of HT effects, namely
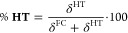
8

**Figure 5:**
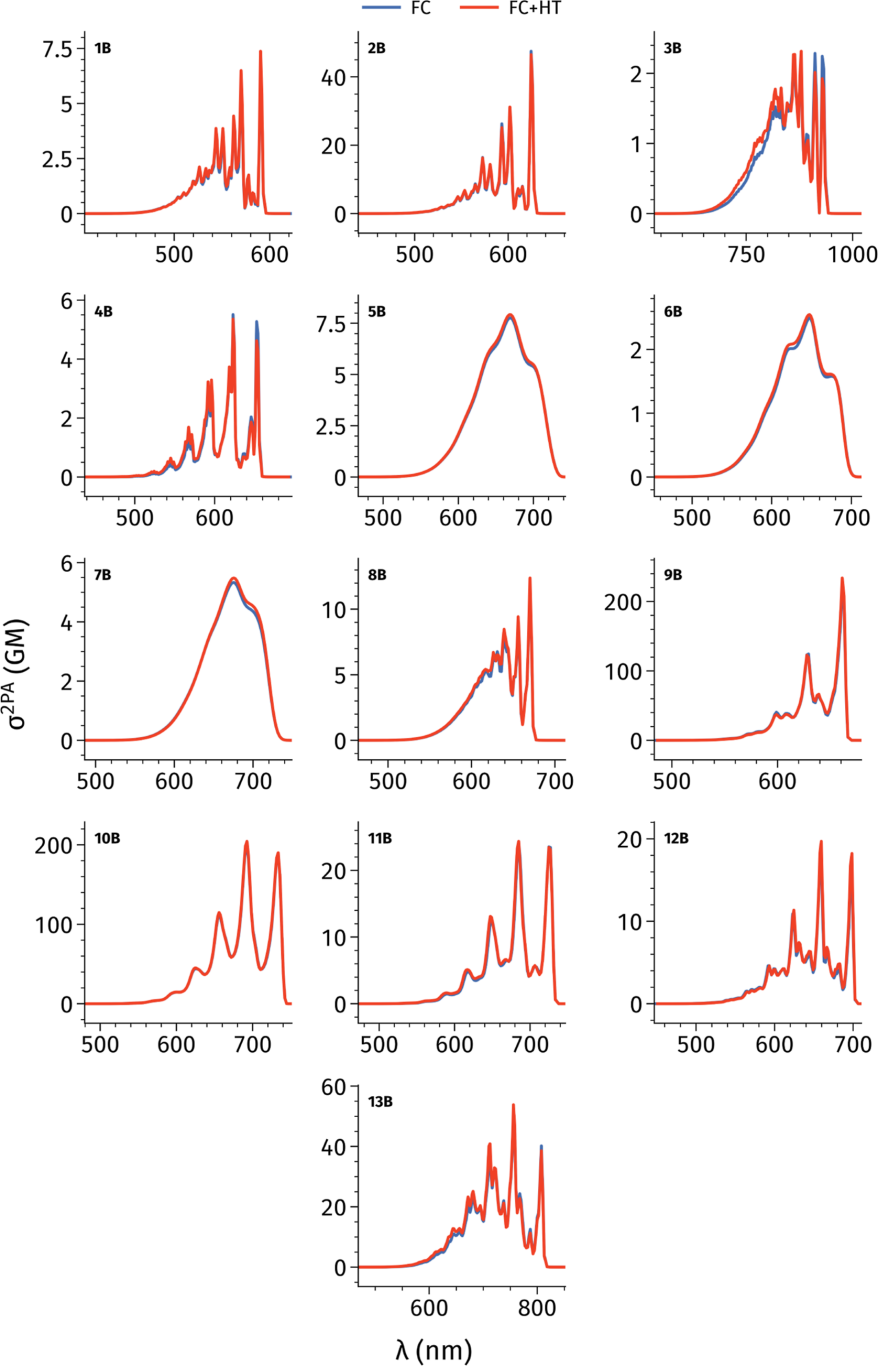
Vibrational LC-BLYP33/6-31+G(d) σ^2PA^ spectra obtained with the TD method, the PES VG model, and a broadening of 0.005 eV, for the different fluorescent dyes computed including FC couplings only (in blue) or both FC and HT terms (in red).

In Figure 4, we observe that HT contributions to the 2PA total intensity have a minimal impact for molecules **1B**–**13B**. The HT term contributes minimally to the total δ value, with contributions ranging from 1.4% to 6.4%. Interestingly, molecules with the largest percentage of HT contributions tend to have low δ^FC^ values, stressing the fact that HT transitions are not predominant for compounds of practical interest. To further explore the effect of HT contributions, Figure 5 presents the vibronic 2PA spectra simulated using both the FC and FC + HT approximations, with the VG model and a homogeneous Gaussian broadening of 0.005 eV.

Figure 5 shows that HT contributions not only have a minor influence on the total intensity of the 2PA π → π* electronic bands, but also that they do not play a significant role in determining the vibronic shape of the bands. The inclusion of HT couplings results in slight variations in the intensity of a few peaks compared to the FC approximation. For instance, in molecule **3B**, we observe a significant reduction in 2PA intensity for high-wavelength modes and an increase for lower-wavelength modes. Conversely, molecule **13B** shows a slight intensity increase in high-wavelength modes. To quantify the impact of HT contributions, we computed vibrationally resolved spectra using a broader line width of 0.100 eV, which is better suited for direct comparisons with experimental data (see Figure S6 in the Supporting Information). We interpolated the peaks from these spectra and compared the results obtained using the FC and FC + HT models. The corresponding data are reported in Table S9 in the Supporting Information. Focusing on the peak positions, we see that the slight increase in low-wavelength peaks identified in Figure 5 leads to small blueshifts in the spectra, an effect being negligible in most cases except for molecule **3B**, for which HT couplings cause a blueshift of 8.7 nm (0.02 eV). Conversely, a few molecules (**1B**, **9B**, **12B**) exhibit slight (less than 2 nm) redshifts when HT effects are included. Including HT effects minimally impacts the intensity of the maximum peaks with variations of less than 1 GM, except for molecules **9B** and **10B**, which show slight enhancement of the 2PA signal by 3.2 GM, 1.3 GM, and 1.1 GM, respectively.

#### 3.2.1 Impact of the Vibronic Model

**Figure 6:**
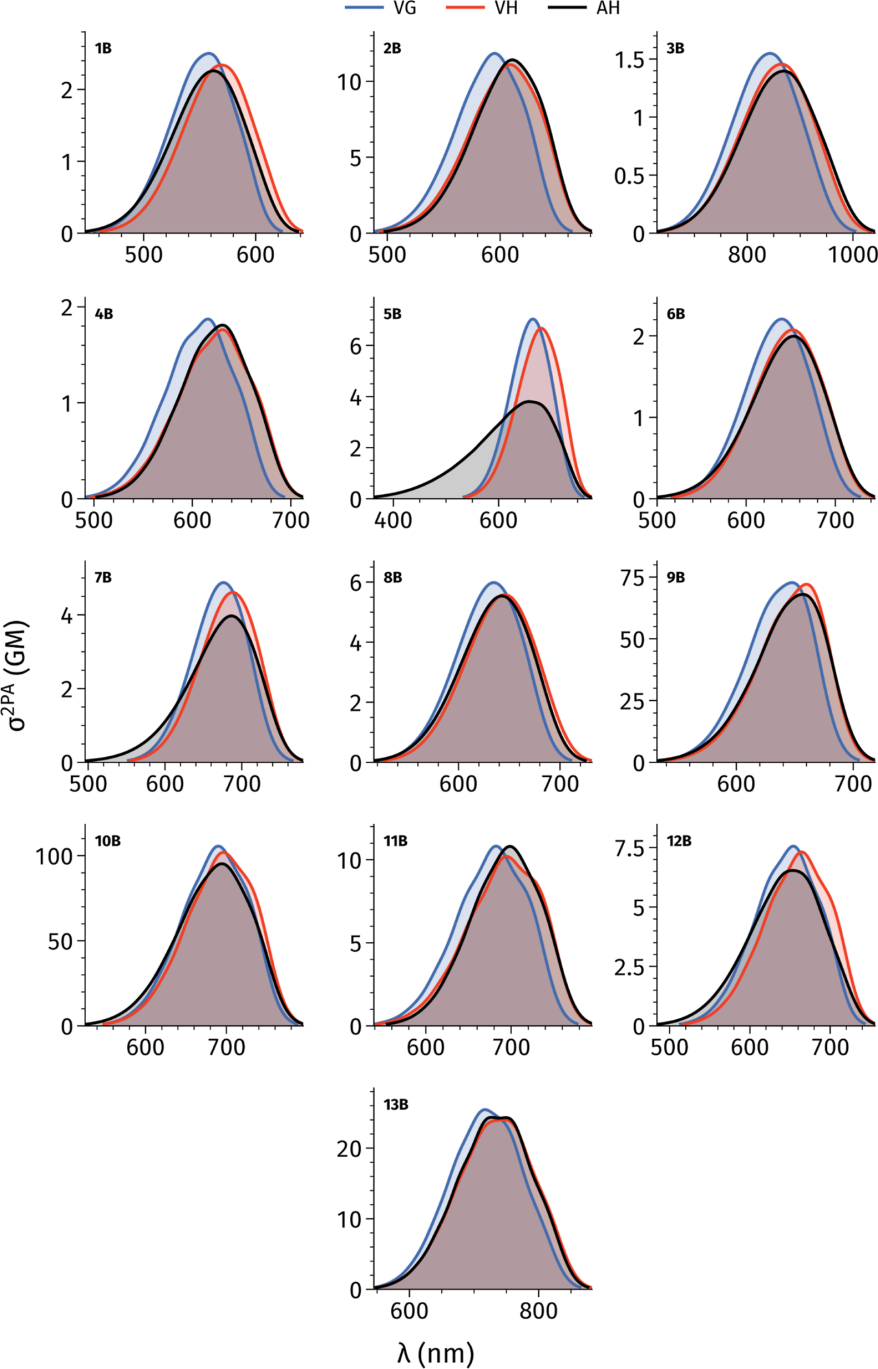
Vibrational LC-BLYP33/6-31+G(d) σ^2PA^ spectra employing the TD method, and the VG, VH, and AH PES models. A Gaussian broadening with a width of 0.100 eV is applied and both FC and HT couplings are included.

For molecules **1B**–**13B** we compared the results obtained with the VG, VH, and AH PES models. To estimate the suitability of the harmonic approximation in the AH framework, we computed the RMSD values between the ground- and excited-state geometries (see Table S11 in the Supporting Information). For the studied molecules the RMSD values remain below 0.17 Å, indicating that the AH approximation is likely suitable for those cases. Figure 6 provides the corresponding vibronic spectra with a 0.100 eV broadening. As above, we interpolated the values of main peaks and identified the corresponding wavelengths, as well as the full-width half maxima (Δν) and the integral surfaces (*I*). These values are listed in Table S10 in the Supporting Information.

By and large, the three methods yield qualitatively similar spectra with relatively minor topological differences. Transition wavelength shifts are observed when moving from the simpler VG model to the more sophisticated VH and AH methods. Specifically, for the VH model a redshift is noted for all the molecules with respect to VG values, ranging from 5.8 to 30.2 nm (0.01 to 0.07 eV) for VH model. Similarly, for AH model we observe a redshift for 11 molecules ranging from 3.8 (0.02 eV) to 26.7 nm (0.05 eV). Globally such a trend is expected.^79−81^ Conversely, for molecules **5B** and **12B**, AH exhibits a modest blueshift of 1.8 nm (0.01 eV) for **12B** and 7.1 nm (0.02 eV) for **5B**. Dye **10B** shows a notable variation in the maximum σ^2PA^ for AH yielding intensities smaller by 10.3 GM, while for VH it is smaller of 3.7 GM. However, these values are less significant in percentage given that the σ^2PA^ for this molecule is ca. 100 GM. Moreover, in molecule **5B** the AH spectra deviates significantly from the other two models, showing a smaller σ^2PA^ of 3.8 GM, compared to 7.0 GM (VG) and 6.7 GM (VH). Additionally, AH produces a broader spectral profile (see below). For the remaining molecules, variations among the three methods are negligible, with VG consistently yielding the largest responses.

Focusing now on the full-width at half-maximum (fwhm) values, we notice that VH and AH tend to produce larger values than VG, a trend previously observed in 1PA spectra.^82^ Although these differences are rather negligible for most molecules, they become significant for molecule **5B**, with fwhm differences of 76.1 nm for AH. This aligns with the intensity changes discussed above and correlates with the larger RMSD observed between ground and excited-state structures, indicating a more pronounced geometry relaxation in the excited state. A few exceptions exist, where VG yields slightly larger fwhm values, specifically for molecules **9B** (relative to VH only) and **11B** (relative to AH), yet these differences remain minimal (<1.4 nm).

## 4 Conclusions

In this work, we systematically assessed the importance of non-Condon effects in simulating 2PA spectra. Using a series of representative molecules, we evaluated the performance of various DFAs against RI-CC2 in reproducing the tensor elements of two-photon transition moments and their first- and second-order derivatives with respect to normal modes. The present findings align with previous studies regarding electronic contributions to two-photon transition strengths,^48,49^ i.e., the global hybrid *meta*-GGA MN15 functional outperforms all the other studied range-separated hybrid, hybrid, and GGA functionals for the investigated molecules. The analysis of the first- and second-order derivatives of second-order transition moment with respect to the normal modes indicate an unsatisfactory accuracy across all DFAs, except for LC-BLYP33 and LC-BLYP47, which deliver an error margin in the range of 40–50% compared to the RI-CC2 reference. In particular, LC-BLYP33 can likely be recommended for its ability to accurately predict the peak positions due to the HT contributions to the 2PA spectrum, though it tends to underestimate the HT contribution to the total intensity of the 2PA π → π* electronic bands. We emphasize that while DFAs for 2PA still fall short of the accuracy achieved by wave function-based methods, they can provide qualitatively reliable results.

Building on these findings, we explored the influence of non-Condon effects on a series of commonly used dyes and fluorescent chromophores within proteins. Across different models of dipole expansion, HT contributions were found to be secondary to their FC counterparts: the largest observed peak shifts are around 9 nm while the intensity changes are trifling. Given the overall insignificant effect of linear Herzberg–Teller term on the shape of the spectra of the investigated fluorophores, one may assume the electrical anharmonicity effects to be negligible. Additionally, we evaluated the AH, VH, and VG vibronic models for simulating the 2PA vibronic spectra, finding that these models produced consistent results for all cases, indicating that the harmonic approximation is generally suitable for such molecular systems. The next logical steps would be to extend this analysis to larger systems and benchmark normal-mode derivatives of second-order transition moments at higher levels of theory, such as CCSD or CC3, which is unfortunately beyond reach at present. To achieve this, further research into the development of analytical gradients for two-photon moments is essential, as it would greatly enhance the efficiency of 2PA spectra calculations, allowing for efficient computations on more complex molecular systems.

## Supporting Information Available

Additional info: benchmark of LC-BLYP variants for two-photon absorption strengths; selection of vibrational modes; performances of density functional approximations in the simulations of two-photon transition strengths/spectra; theoretical and experimental one-photon spectra; comparison of two-photon absorption spectra for different vibronic models; Cartesian coordinates of molecules (PDF)
