# Supplementary material for: Simulations of Two-Photon Absorption Spectra of Fluorescent Dyes: The Impact of Non-Condon Effects
Source: J Chem Theory Comput. 2025 Mar 26;21(7):3587–99. doi: 10.1021/acs.jctc.4c01545 (PMC11983704; doi:10.1021/acs.jctc.4c01545)
Supplement: Supplementary file 1 — ct4c01545_si_001.pdf [file ct4c01545_si_001.pdf]

Supporting Information: Simulations of Two-Photon Absorption Spectra of Fluorescent Dyes: The Impact of Non-Condon Effects

Rudraditya Sarkar,[†,‡,¶] Carmelo Naim,[§,¶] Karan Ahmadzadeh,[‖] Robert Zaleśny,[*,⊥] Denis Jacquemin,[*,§,#] and Josep M. Luis[*,†]

†*Institute of Computational Chemistry and Catalysis and Department of Chemistry, University of Girona, Campus de Montilivi, 17003, Girona, Catalonia, Spain.*

‡*Department of Chemistry, School of Science, Gandhi Institute of Technology and Management (GITAM), Hyderabad 502329, India*

¶*These authors contributed equally to this work*

§*Nantes Université, CNRS, CEISAM UMR 6230, F-44000 Nantes, France*

‖*Hefei National Research Center for Physical Sciences at the Microscale, University of Science and Technology of China, Hefei, Anhui 230026, China*

⊥*Faculty of Chemistry, Wrocław University of Science and Technology, Wyb. Wyspiańskiego 27, PL–50370 Wrocław, Poland*

#*Institut Universitaire de France (IUF), F-75005, Paris, France*

E-mail: robert.zalesny@pwr.edu.pl; Denis.Jacquemin@univ-nantes.fr; Josepm.Luis@udg.edu

Contents

S1 Benchmark systems **S3**

S1.1 Benchmark of LC-BLYP variants for 2PA strengths S3

S1.2 Vibrational modes included in the study S4

S1.3 Performances of DFAs on the two-photon transition moments S6

S1.4 Performances of DFAs on the 2PA spectra S9

S2 Fluorescent molecules **S10**

S2.1 1PA theoretical and experimental spectra S10

S2.2 Comparison of 2PA spectra for different vibronic models S12

S2.3 Optimized Geometries S17

References **S44**

S1 Benchmark systems

S1.1 Benchmark of LC-BLYP variants for 2PA strengths

Table S1: Error analysis for the 2PA strength (δ^{2PA}) of first 46 molecules of Ref.[S1] The errors are calculated with respect to the data obtained from the RI-CC2 level of theory. The data are arranged following small to large MAE.

DFA	MAE	SDE	RMSE	MAPE
LC-BLYP (μ=0.100)	13605	20257	20083	42
LC-BLYP (μ=0.125)	13930	22736	25167	31
LC-BLYP (μ=0.150)	18817	26846	32300	36
LC-BLYP (μ=0.075)	20811	26091	28210	71
LC-BLYP (μ=0.175)	23480	30104	37910	44
LC-BLYP (μ=0.200)	26821	32573	41920	53
LC-BLYP (μ=0.050)	30008	36719	42676	106
OT-LC-BLYP	30498	33909	45331	63
LC-BLYP (μ=0.300)	33434	37304	49792	69
LC-BLYP (μ=0.330)	34410	38013	50967	71
LC-BLYP (μ=0.400)	36182	39097	52957	76
LC-BLYP (μ=0.025)	40803	53315	62862	133

S1.2 Vibrational modes included in the study

Table S2: Characterization of the selected vibrational modes for molecules **1A-6A**. The $\frac{\partial S_{xx}}{\partial Q}$ values are estimated at LC-BLYP47/6-31+G(d) level and the values of $\frac{\partial S_{xx}}{\partial Q}$ is given in a.u.

Molecule	vibrational mode	mode assignment	$\frac{\partial S_{xx}}{\partial Q}$
	ν_{42}	in-plane-bending (Scissoring)	0.55757
**1A**	ν_{57}	C-C stretching + in-plane-bending (Rocking)	-0.70703
	ν_{58}	C-C stretching + in-plane-bending (Scissoring)	1.12995
	ν_{65}	N-O + N-C stretching (Asymmetric)	3.00347
**2A**	ν_{67}	in-plane-bending (Rocking)	-0.99514
	ν_{71}	benzene C-C stretching + in-plane-bending (Scissoring)	-1.41776
	ν_{53}	N-O + N-C stretching (Asymmetric)	2.45037
**3A**	ν_{60}	benzene C-C stretching + in-plane-bending (Scissoring/benzene)	-1.28857
	ν_{61}	benzene C-C stretching + in-plane-bending (Scissoring/benzene)	-0.97438
	ν_{62}	in-plane H-N-H bending (Rocking) + benzene C-C stretching + in-plane-bending (Scissoring/benzene)	-1.76356
**4A**	ν_{64}	C-C (triple bond) stretching	1.04718
	ν_{65}	C-N (triple bond) stretching	-1.13126
	ν_{61}	N-O stretching	-3.19250
**5A**	ν_{63}	in-plane-bending (Rocking/benzene)	1.04560
	ν_{67}	in-plane bending (Rocking/benzene) + C-C stretching	1.67019
	ν_{47}	in-plane-bending (Scissoring/benzene)	1.26334
**6A**	ν_{59}	C-NO_2 stretching	-3.48942
	ν_{65}	in-plane-bending (Scissoring/benzene)	2.24611
	ν_{77}	N-H stretching (Symmetric)	-0.70561

Figure S1: Atomic displacements for the selected subset of vibrational normal modes for molecules **1A**-**6A** as determined at the LC-BLYP47/6-31+G(d) level of theory.

(a) **1A** (ν_{42}), $\frac{\partial S_{xx}}{\partial Q_\nu}$=0.558

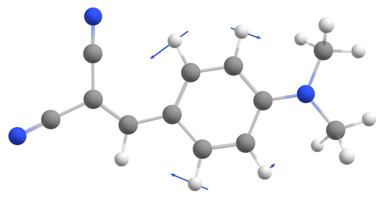

(b) **1A** (ν_{57}), $\frac{\partial S_{xx}}{\partial Q_\nu}$=-0.707

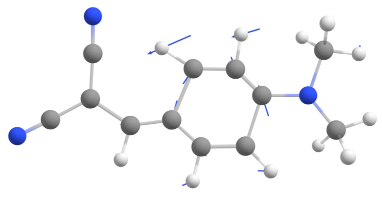

(c) **1A** (ν_{58}), $\frac{\partial S_{xx}}{\partial Q_\nu}$=1.130

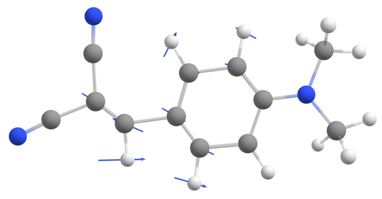

(d) **2A** (ν_{65}), $\frac{\partial S_{xx}}{\partial Q_\nu}$=3.004

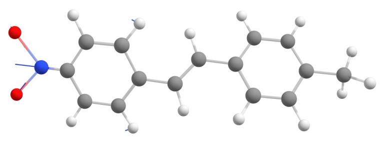

(e) **2A** (ν_{67}), $\frac{\partial S_{xx}}{\partial Q_\nu}$=-0.995

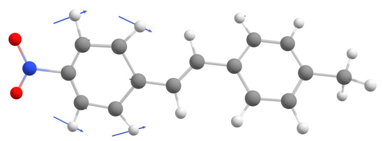

(f) **2A** (ν_{71}), $\frac{\partial S_{xx}}{\partial Q_\nu}$=-1.418

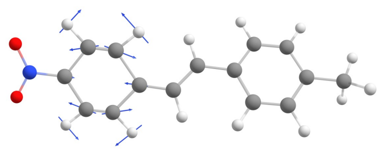

(g) **3A** (ν_{53}), $\frac{\partial S_{xx}}{\partial Q_\nu}$=2.450

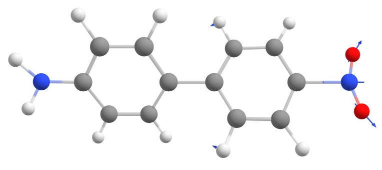

(h) **3A** (ν_{60}), $\frac{\partial S_{xx}}{\partial Q_\nu}$=-1.289

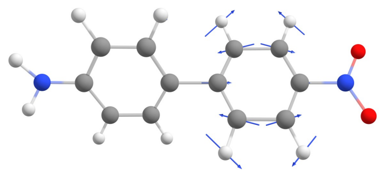

(i) **3A** (ν_{61}), $\frac{\partial S_{xx}}{\partial Q_\nu}$=-0.974

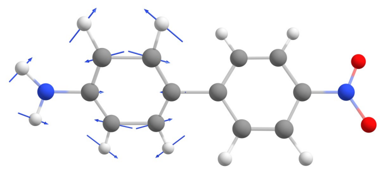

(j) **4A** (ν_{62}), $\frac{\partial S_{xx}}{\partial Q_\nu}$=-1.764

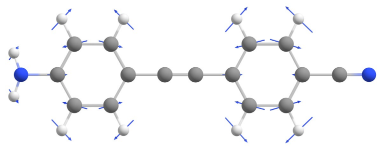

(k) **4A** (ν_{64}), $\frac{\partial S_{xx}}{\partial Q_\nu}$=1.047

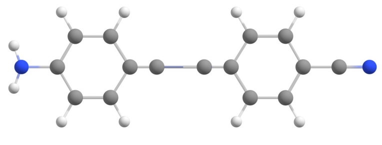

(l) **4A** (ν_{65}), $\frac{\partial S_{xx}}{\partial Q_\nu}$=-1.131

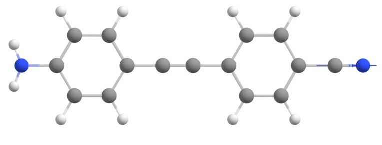

(m) **5A** (ν_{61}), $\frac{\partial S_{xx}}{\partial Q_\nu}$=-3.193

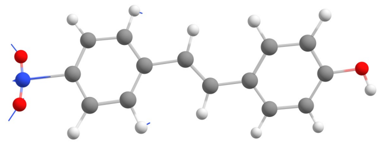

(n) **5A** (ν_{63}), $\frac{\partial S_{xx}}{\partial Q_\nu}$=1.046

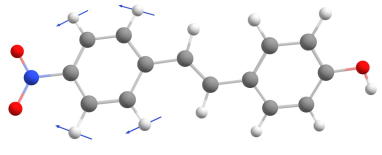

(o) **5A** (ν_{67}), $\frac{\partial S_{xx}}{\partial Q_\nu}$=1.670

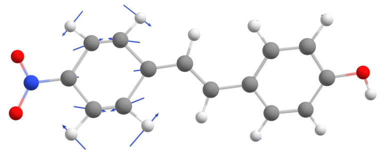

(p) **6A** (ν_{47}), $\frac{\partial S_{xx}}{\partial Q_\nu}$=1.263

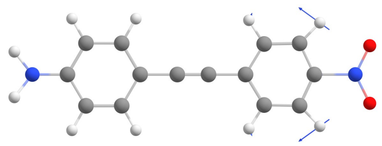

(q) **6A** (ν_{59}), $\frac{\partial S_{xx}}{\partial Q_\nu}$=-3.489

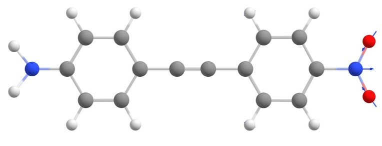

(r) **6A** (ν_{65}), $\frac{\partial S_{xx}}{\partial Q_\nu}$=2.246

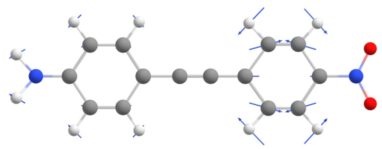

(s) **6A** (ν_{77}), $\frac{\partial S_{xx}}{\partial Q_\nu}$=-0.706

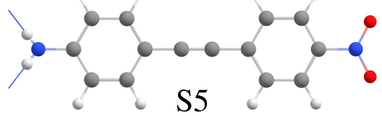

S1.3 Performances of DFAs on the two-photon transition moments

Table S3: Electronic S_{xx} values for **1A–6A** at the LC-BLYP47/6-31+G(d) ground state geometry. All calculations were performed using cc-pVDZ basis set.

	**1A**	**2A**	**3A**	**4A**	**5A**	**6A**
RI-CC2	302.6	242.5	277.3	355.0	310.6	432.9
MN15	147.6	249.7	263.2	281.0	296.0	415.3
PBE	132.8	432.8	393.3	459.7	494.5	630.5
PBE0	144.4	328.3	317.5	356.9	385.5	521.2
BLYP	132.7	434.6	395.6	457.9	496.1	632.9
LC-BLYP10	139.9	328.6	326.7	363.3	391.6	513.4
LC-BLYP33	152.5	152.4	181.7	190.7	190.5	258.9
LC-BLYP47	148.5	133.3	157.5	162.6	164.6	221.2
B3LYP	142.4	353.9	334.8	375.9	412.3	550.6

Table S4: Statistical analysis of S_{xx} (a.u.) performed for molecules **1A**-**6A**. The errors are calculated with respect to the data obtained from the RI-CC2 level of theory. The data are arranged according to increasing MAE. The cc-pVDZ basis set was employed.

DFA	MAE	SDE	RMSE	MAX AE	MAPE
MN15	47.06	60.51	71.03	155.02	15
PBE0	74.90	94.32	88.91	158.17	25
LC-BLYP10	78.03	95.97	90.78	162.70	26
B3LYP	94.90	105.53	104.90	160.20	31
LC-BLYP33	132.34	35.67	136.29	173.93	41
LC-BLYP47	155.52	40.09	159.77	211.68	48
PBE	160.42	139.82	164.53	197.65	52
BLYP	161.46	140.61	165.73	200.04	52

Table S5: $\frac{\partial S_{xx}}{\partial Q}$ values for the selected normal modes of molecule **1A-6A** obtained at the RI-CC2 level of theory and different density functional using cc-pVDZ basis set.

	**1A**			**2A**			**3A**		
	ν_{42}	ν_{57}	ν_{58}	ν_{65}	ν_{67}	ν_{71}	ν_{53}	ν_{60}	ν_{61}
RI-CC2	1.46	-0.76	1.90	3.51	-1.43	-1.53	2.94	-1.51	-0.69
BLYP	1.27	-1.19	2.87	1.80	-1.27	1.66	1.45	1.72	0.83
PBE	1.26	-1.17	2.81	1.82	-1.29	1.59	1.46	1.67	0.84
PBE0	1.30	-1.24	2.89	3.29	-1.66	0.71	2.28	-0.21	-0.45
B3LYP	1.32	-1.26	2.96	3.11	-1.59	0.97	2.29	0.01	-0.33
LC-BLYP10	1.31	-1.21	2.82	1.21	-1.27	0.93	1.74	0.13	-0.16
LC-BLYP33	0.70	-0.80	1.43	2.37	-0.92	-1.05	1.90	-0.93	-0.74
LC-BLYP47	0.52	-0.66	1.07	2.26	-0.80	-1.17	1.92	-1.07	-0.80
LC-BLYP47^{+}	0.56	-0.71	1.13	3.00	-1.00	-1.42	2.45	-1.29	-0.97
MN15	1.09	-1.11	2.43	3.63	-1.61	-0.11	2.82	-0.38	-0.55

	**4A**			**5A**			**6A**			
	ν_{62}	ν_{64}	ν_{65}	ν_{61}	ν_{63}	ν_{67}	ν_{47}	ν_{59}	ν_{65}	ν_{77}
RI-CC2	-2.73	1.42	-1.81	-6.32	1.80	1.14	1.89	-3.93	3.07	-1.78
BLYP	1.46	-6.09	-2.65	-1.62	1.23	-2.16	-1.31	-1.41	-3.52	-1.15
PBE	1.49	-6.07	-2.60	-1.66	1.25	-2.10	-1.29	-1.45	-3.44	-1.16
B3LYP	-0.08	-4.78	-2.65	-3.09	1.61	-0.76	-0.11	-3.56	-0.74	-1.33
PBE0	-0.35	-4.29	-2.52	-3.30	1.70	-0.48	0.11	-3.81	-0.30	-1.36
LC-BLYP10	0.15	-4.76	-2.31	-2.38	1.44	-0.85	-0.19	-2.80	-0.86	-1.21
LC-BLYP33	-1.52	0.33	-1.07	-2.50	0.96	1.35	1.01	-2.88	1.75	-0.77
LC-BLYP47	-1.60	0.82	-0.89	-2.35	0.84	1.46	1.07	-2.72	1.95	-0.71
LC-BLYP47^{+}	-1.76	1.05	-1.13	-3.19	1.05	1.67	1.26	-3.49	2.25	-0.71
MN15	-1.13	-2.44	-1.96	-6.32	1.93	-0.77	0.68	-4.12	0.76	-1.21

$^{+}$computed with 6-31+G(d,p) basis set

Table S6: $\frac{\partial^2 S_{xx}}{\partial Q^2}$ values and corresponding percentage numerical error in parenthesis for the selected normal modes of molecule 1A and 4A obtained at the RI-CC2 level of theory and different density functional using cc-pVDZ basis set.

	**1A**			**4A**		
	ν_{42}	ν_{57}	ν_{58}	ν_{62}	ν_{64}	ν_{65}
RI-CC2	-36.9 (0.0)	-29.0 (0.0)	-212.7 (0.1)	21.7 (0.0)	-151.4 (0.1)	34.1 (0.0)
BLYP	-6.1 (0.0)	-9.8 (0.0)	-31.8 (0.0)	-159.8 (0.0)	-384.7 (0.1)	4.6 (0.0)
PBE	-5.8 (0.0)	-9.3 (0.0)	-28.4 (0.0)	-156.3 (0.0)	-365.7 (0.0)	4.0 (0.0)
PBE0	-9.4 (0.0)	-11.7 (0.0)	-73.2 (0.0)	-104.8 (0.1)	-355.9 (0.0)	34.4 (0.0)
B3LYP	-8.7 (0.0)	-11.5 (0.0)	-67.4 (0.0)	-119.4 (0.0)	-402.2 (1.3)	35.5 (3.4)
CAMB3LYP	-16.8 (0.0)	-13.2 (0.0)	-114.3 (0.0)	-34.5 (0.0)	-196.4 (0.0)	22.5 (0.0)
LC-BLYP10	-7.0 (0.0)	-10.3 (0.0)	-53.1 (0.0)	-101.9 (0.0)	-317.9 (0.0)	24.1 (0.0)
LC-BLYP33	-21.2 (0.0)	-18.6 (0.0)	-134.7 (0.0)	-0.7 (0.0)	-140.5 (0.0)	11.8 (0.0)
LC-BLYP47	-25.2 (0.0)	-25.0 (0.0)	-151.7 (0.0)	9.8 (0.0)	-122.7 (0.0)	6.7 (0.0)
MN15	-14.3 (0.0)	-13.4 (0.0)	-100.0 (0.0)	-59.5 (0.0)	-235.5 (0.0)	28.9 (0.0)

Table S7: Error analysis of the excitation energy (in eV) of the first bright state of molecule **1A**-**6A**. The errors are calculated with respect to the data obtained at the RI-CC2 level of theory. The data are arranged following small to large MAE. cc-pVDZ basis set is employed for all quantum chemical calculations.

DFA	MAE	SDE	RMSE	MAX AE	MAPE
LC-BLYP33	0.09	0.13	0.14	0.29	2
LC-BLYP47	0.23	0.14	0.27	0.45	6
MN15	0.40	0.23	0.43	0.52	11
PBE0	0.66	0.34	0.73	0.93	15
B3LYP	0.80	0.38	0.87	1.13	19
LC-BLYP10	0.89	0.34	0.95	1.25	21
PBE	1.35	0.51	1.43	1.85	32
BLYP	1.37	0.52	1.45	1.87	32

S1.4 Performances of DFAs on the 2PA spectra

Figure S2: The HT and FC+HT spectra for molecule **2A** where the $\frac{\partial S_{xx}}{\partial Q}$ values for some selected normal mode (see Fig. 2) are obtained at the CC2/cc-pVDZ, LC-BLYP33/cc-pVDZ and BLYP/cc-pVDZ levels of theory, while the values of the FC factors and the rest of the $\frac{\partial S_{xx}}{\partial Q}$ derivatives are obtained from LC-BLYP47/6-31+G(d) level.

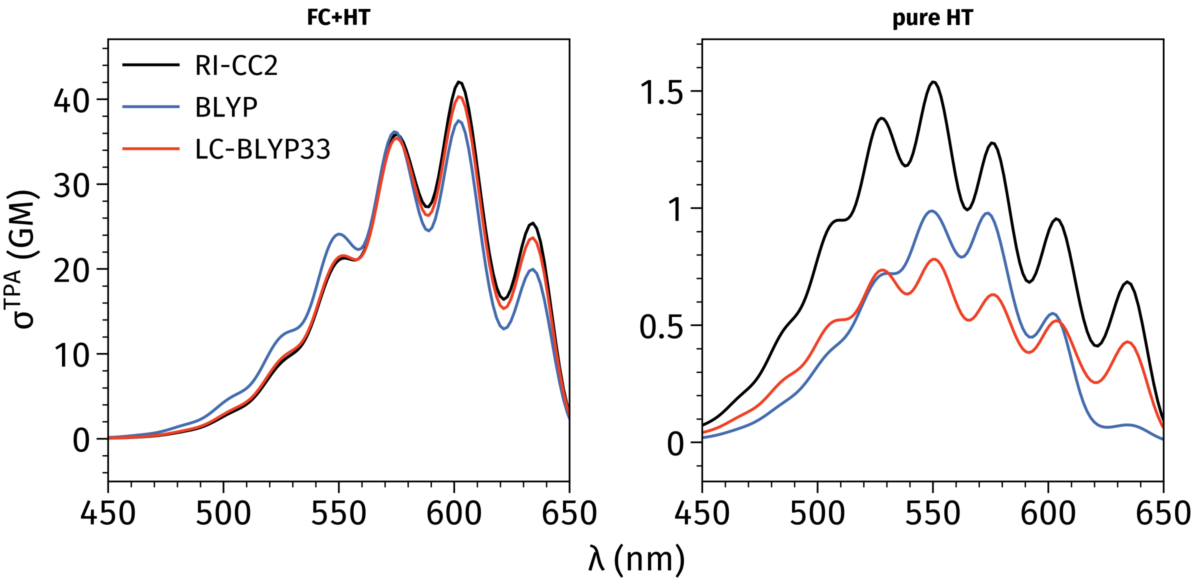

S2 Fluorescent molecules

S2.1 1PA theoretical and experimental spectra

Table S8: Vertical transition energies (ΔE) and oscillator strengths (f) for molecules in set B, calculated at the LC-BLYP33/6-31+G(d) level in vacuum. The solvents used and the corresponding references for the experimental spectra shown in Fig. S3 are also provided. For molecules 2B and 7B, experimental spectra of similar compounds are reported, as detailed in the footnote.

	Theo. vacuum		Exp. data	
Molecule	ΔE[eV]	f	Solvent	Ref.
**1B**	4.5	0.3	Chloroform	S2
**2B**	4.2	0.5	Toluene	S3[a]
**3B**	3.0	0.2	Chloroform	S4
**4B**	3.9	0.1	Cyclohexane	S5
**5B**	3.7	0.2	n-hexane	S6
**6B**	4.1	0.3	Toluene	S7[b]
**7B**	3.8	0.3	DCM	S8
**8B**	4.0	0.1	Chloroform	S9
**9B**	3.9	1.1	Chloroform	S10
**10B**	3.6	1.4	Chloroform	S10
**11B**	3.7	0.6	Protein	S11
**12B**	3.9	0.7	Protein	S11
**13B**	3.5	0.8	Protein	S12

[a]Ethyl groups instead of Methyl groups linked to the Nitrogen
[b]Butyl group instead of Hydrogen linked to the Nitrogen

Figure S3: Experimental and computational vibronic one-photon absorption (OPA) spectra for molecules in set B. The computational spectra are aligned with the experimental peaks for clarity. References for the experimental data and details of the solvents used are provided in Table S8. Theoretical spectra were calculated at the LC-BLYP33/6-31+G(d) level of theory in vacuum with a TD-DFT formulation, employing the VG model. Both Franck-Condon (FC, red) and Franck-Condon plus Herzberg-Teller (FC+HT, blue) expansions of the dipole moments are shown, with a broadening of 0.100 eV. Note that, except for molecule **13B**, the FC and FC+HT band shapes overlap.

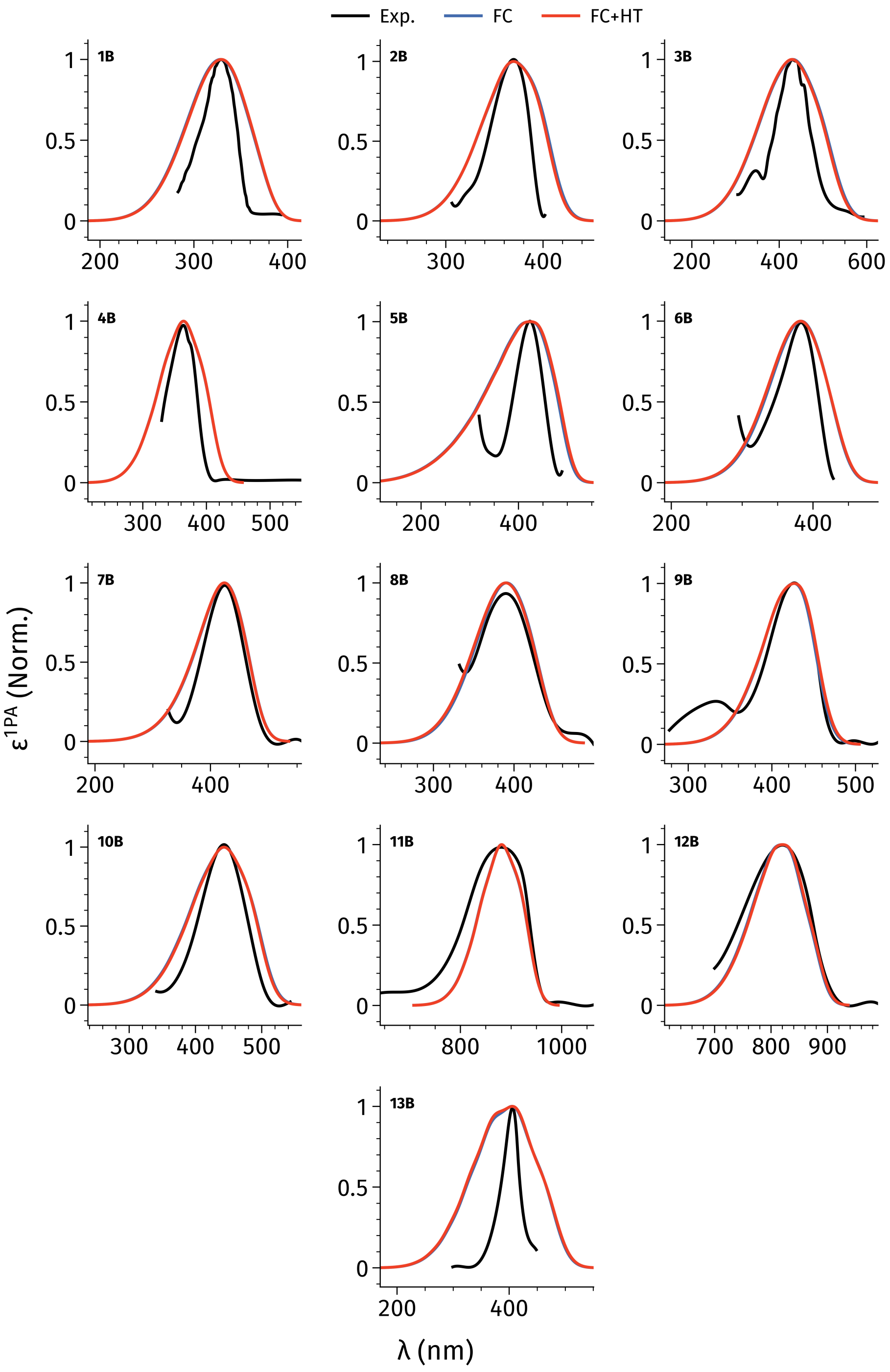

S2.2 Comparison of 2PA spectra for different vibronic models

Table S9: Wavelengths and two-photon absorption intensities for FC (λ^{FC}, σ^{FC}) and FC+HT ($\lambda^{\mathrm{FC+HT}}$,$\sigma^{\mathrm{FC+HT}}$) extrapolated from the spectra in Fig. S6. Relative differences among the two quantities are also reported.

Molecule	$\lambda^{\mathrm{FC+HT}}$ [nm]	λ^{FC} [nm]	$\Delta\lambda$ [nm]	$\sigma^{\mathrm{FC+HT}}$ [GM]	σ^{FC} [GM]	$\Delta\sigma$ [GM]
**1B**	558.5	557.2	1.3	2.5	2.4	0.2
**2B**	594.6	594.6	0.0	11.8	11.7	0.1
**3B**	843.4	852.1	-8.7	1.5	1.5	0.1
**4B**	615.3	618.4	-3.1	1.9	1.9	-0.0
**5B**	664.8	664.8	0.0	7.0	6.9	0.2
**6B**	640.7	640.7	0.0	2.2	2.1	0.1
**7B**	675.7	675.7	0.0	4.9	4.7	0.1
**8B**	634.2	635.8	-1.6	6.0	5.7	0.3
**9B**	647.4	645.8	1.7	72.7	69.5	3.2
**10B**	690.7	690.7	0.0	105.6	104.5	1.1
**11B**	683.1	683.1	0.0	10.8	10.6	0.2
**12B**	654.3	652.5	1.7	7.6	7.1	0.5
**13B**	716.7	720.8	-4.2	25.4	24.7	0.7

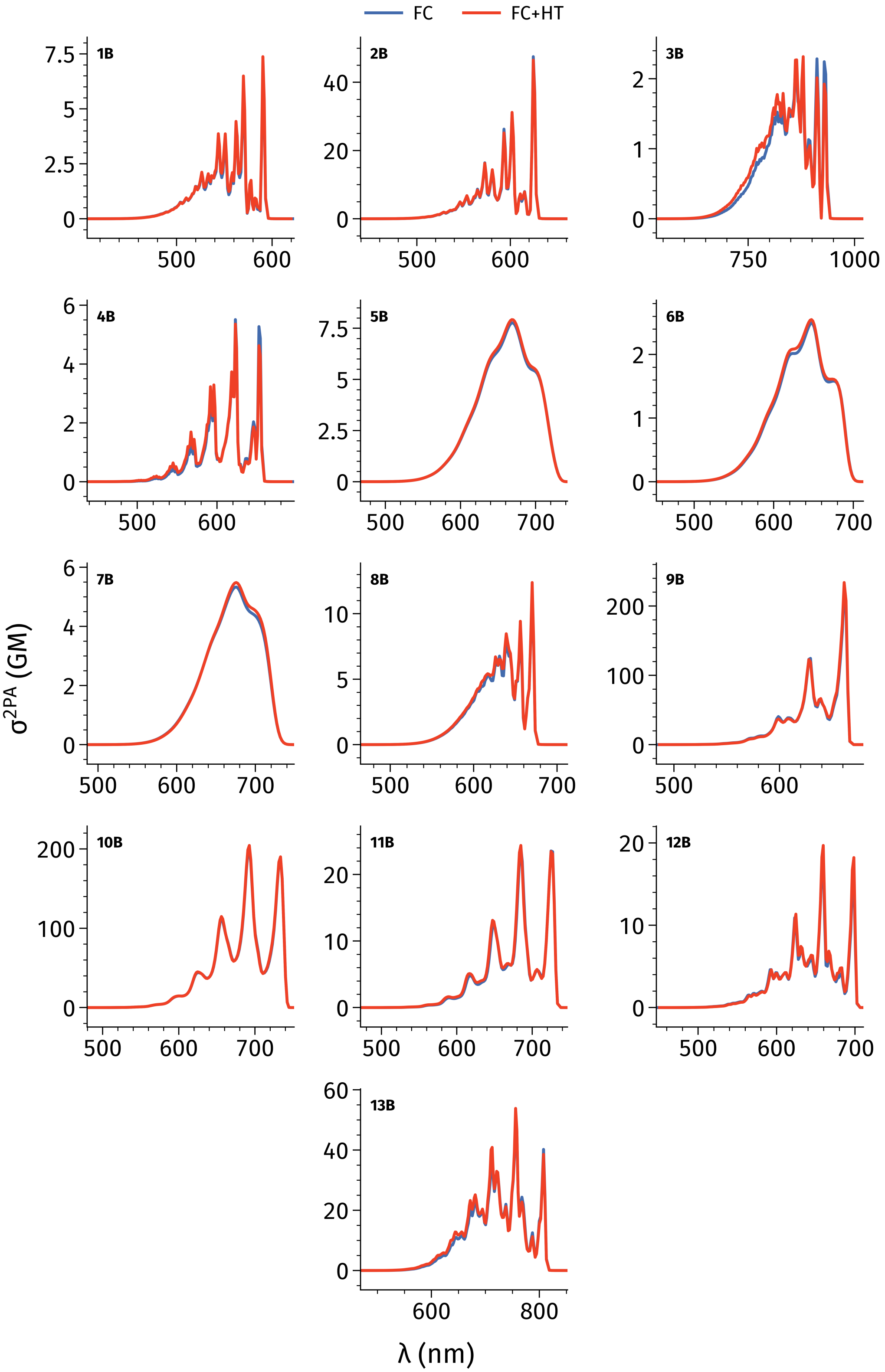

Figure S4: Vibrational σ^{TPA} spectra employing the TD method, VH model, with a broadening of 0.005 eV for the different test fluorescent dyes computing including only FC couplings (in blue) and both FC and HT ones (in red).

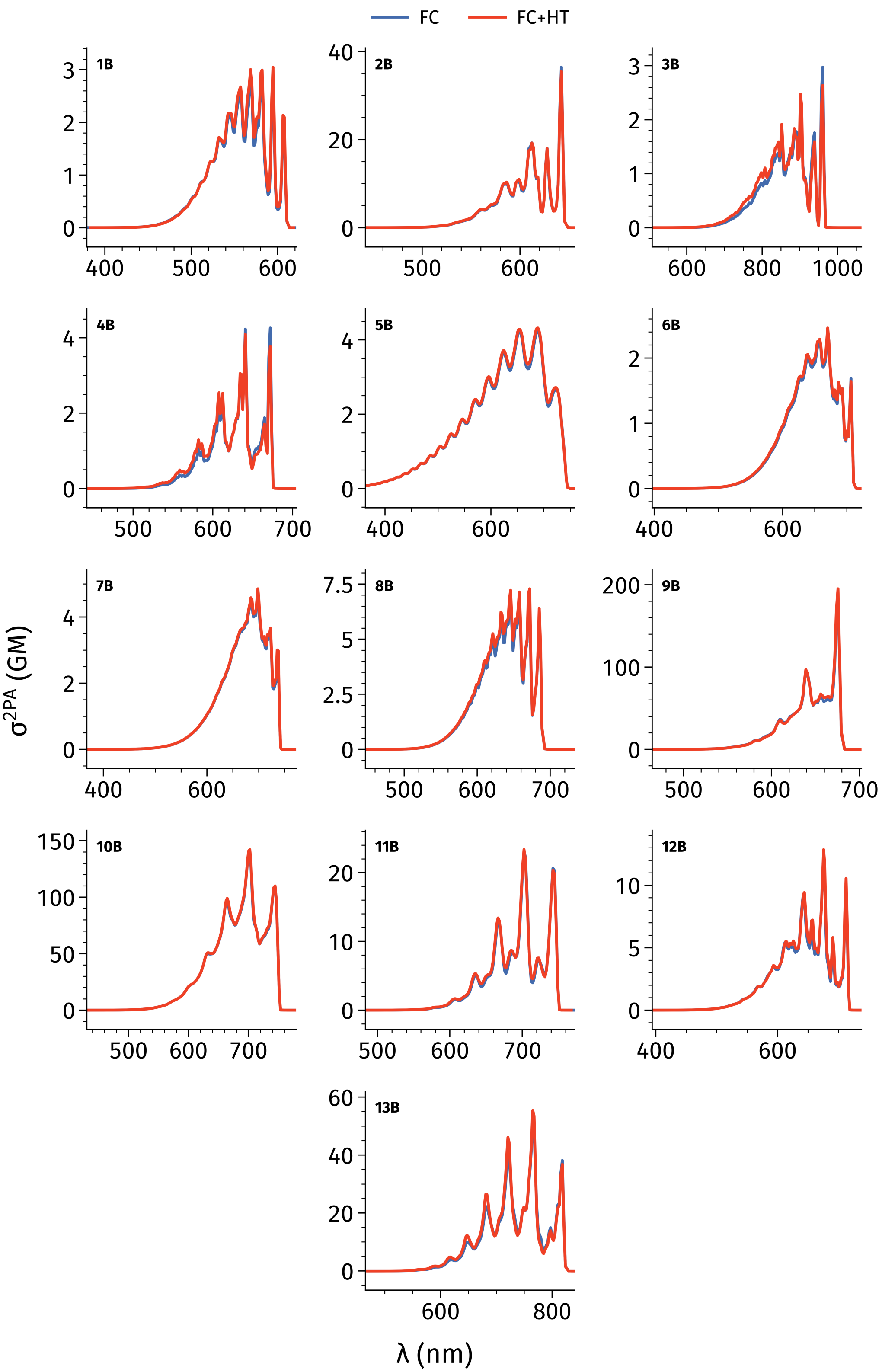

Figure S5: Vibrational σ^{TPA} spectra employing the TD method, AH model, with a broadening of 0.005 eV for the different test fluorescent dyes computing including only FC couplings (in blue) and both FC and HT ones (in red).

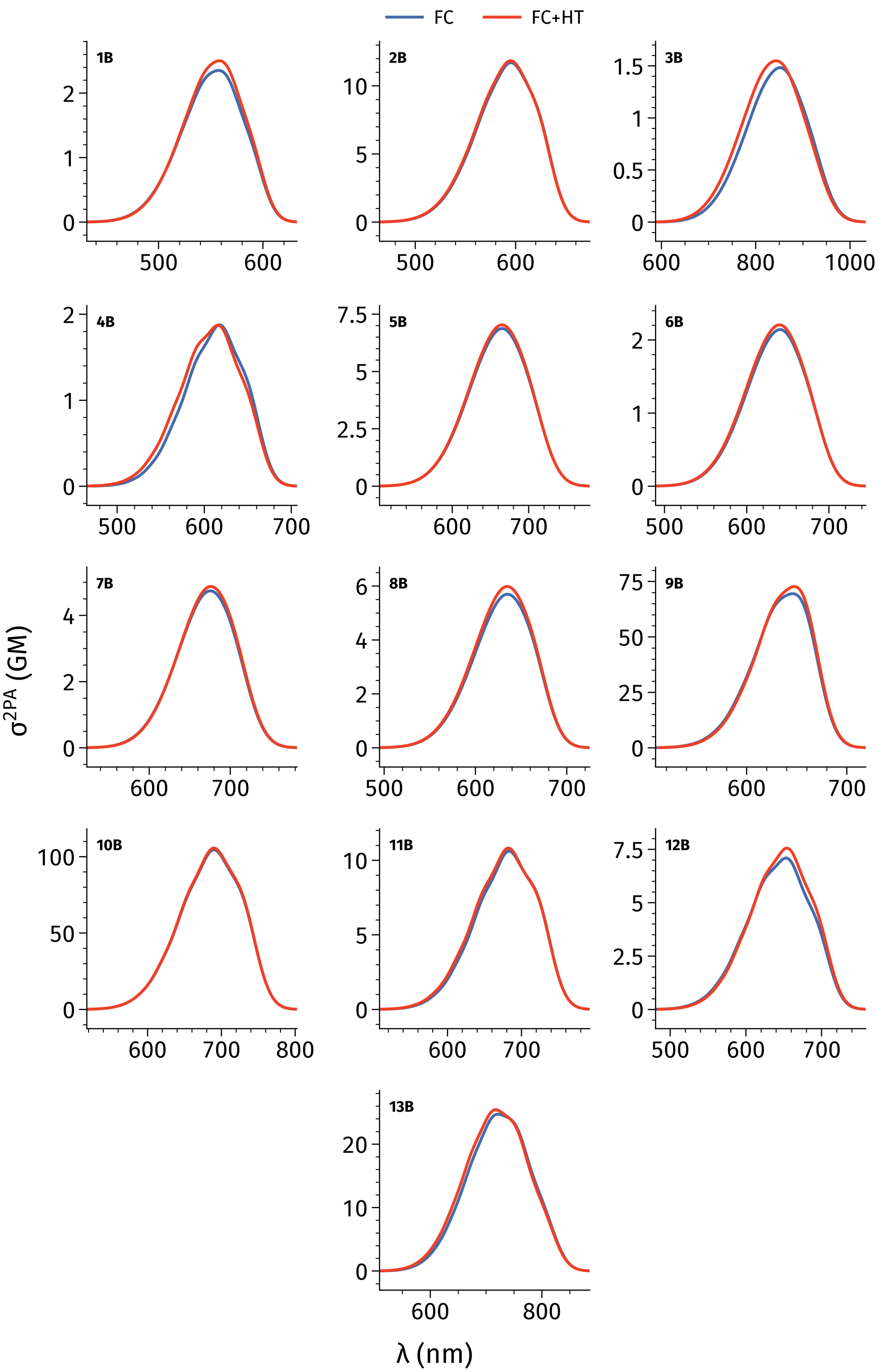

Figure S6: Vibrational $\sigma^{2\text{PA}}$ spectra employing the TD method, VG model, with a broadening of 0.100 eV for the different test fluorescent dyes computed including only FC couplings (in blue) and both FC and HT ones (in red).

Table S10: Transition wavelengths (λ), full width half maximum ($\Delta\nu$), two-photon absorption cross-sections (σ^{2PA}) for VG, VH and AH models extrapolated from the spectra in Fig. 5.

Molecule	λ^{VG}	λ^{VH}	λ^{AH}	$\Delta\nu^{VG}$	$\Delta\nu^{VH}$	$\Delta\nu^{AH}$	σ^{VG}	σ^{VH}	σ^{AH}	I^{VG}	I^{VH}	I^{AH}
		[nm]			[nm]			[GM]			[nm·GM]	
**1B**	558.5	570.0	562.3	74.1	78.7	81.6	2.5	2.3	2.3	187.8	187.8	187.8
**2B**	594.6	609.3	610.8	76.2	81.3	79.1	11.8	11.1	11.4	913.3	913.2	913.2
**3B**	843.4	864.0	870.1	163.5	174.0	181.2	1.5	1.5	1.4	257.4	257.4	257.3
**4B**	615.3	631.0	631.0	91.2	95.8	92.6	1.9	1.8	1.8	168.6	168.6	168.6
**5B**	664.8	681.2	657.7	98.8	103.1	174.9	7.0	6.7	3.8	701.7	701.9	700.9
**6B**	640.7	652.5	652.5	91.3	98.1	99.5	2.2	2.1	2.0	205.8	205.8	205.7
**7B**	675.7	688.8	686.9	91.0	94.0	105.3	4.9	4.6	4.0	447.3	447.2	446.9
**8B**	634.2	645.8	642.4	82.0	87.9	87.0	6.0	5.6	5.5	497.1	497.2	497.2
**9B**	647.4	659.5	656.0	70.5	69.7	74.6	72.7	72.1	68.0	5282.7	5281.5	5281.6
**10B**	690.7	696.5	694.6	105.0	109.0	115.9	105.6	101.9	95.3	11158.4	11156.5	11155.4
**11B**	683.1	694.6	698.5	106.4	110.7	105.1	10.8	10.2	10.8	1146.7	1146.7	1146.5
**12B**	654.3	664.8	652.5	103.5	107.4	114.7	7.6	7.3	6.5	777.5	777.4	777.4
**13B**	716.7	746.9	727.2	143.1	154.4	149.1	25.4	24.0	24.3	3733.6	3734.2	3733.4

S2.3 Optimized Geometries

Table S11: RMSD values between between the ground- and excited-state equilibrium geometries for molecules **1B-13B**, ordered by RMSD value.

Molecule	RMSD (Å)	Molecule	RMSD (Å)
**2B**	0.02	**10B**	0.09
**4B**	0.03	**7B**	0.11
**8B**	0.04	**1B**	0.12
**3B**	0.04	**5B**	0.16
**13B**	0.05	**2B**	0.17
**9B**	0.07		
**6B**	0.08		
**11B**	0.08		

1B-GS

C	-2.3809730	0.7578420	0.0000000
C	0.0000000	0.3898340	0.0000000
C	-0.1526520	-0.9970210	0.0000000
C	-1.4951650	-1.5189980	0.0000000
C	-2.5538810	-0.6906140	0.0000000
H	1.3191810	2.0820010	0.0000000
C	1.2526170	0.9922960	0.0000000
C	1.0072610	-1.7861460	0.0000000
H	-1.6361020	-2.6028310	0.0000000
H	-3.5850070	-1.0400640	0.0000000
C	2.2603550	-1.2144030	0.0000000
C	2.3771480	0.1822430	0.0000000
H	0.9046500	-2.8736460	0.0000000
H	3.1687650	-1.8166360	0.0000000
O	-3.2683500	1.5764920	0.0000000
O	-1.0781220	1.2217130	0.0000000
O	3.6372260	0.6889430	0.0000000
H	3.6142190	1.6638000	0.0000000

1B-ES1

C	-2.4648490	-0.5020740	-0.0297810
C	-0.0533780	-0.4070990	0.1038640
C	-0.0782160	1.0495940	0.0726690
C	-1.3238270	1.6914280	0.0380600
C	-2.4848070	0.9049460	-0.1232530
H	1.0976220	-2.2073860	0.0178120
C	1.1386230	-1.1164050	0.0049100
C	1.1830840	1.7077340	0.0290890
H	-1.3790500	2.7793680	0.0160050
H	-3.4600660	1.3549810	-0.3021100
C	2.3548980	0.9997280	-0.0231300
C	2.3342880	-0.4222250	-0.0454890
H	1.1983340	2.7987590	0.0279810
H	3.3246070	1.4976570	-0.0562010
O	-3.3546550	-1.3201930	-0.1554040
O	-1.1687290	-1.1082180	0.2955800
O	3.5413660	-1.0287620	-0.1076090
H	3.4398140	-1.9997620	-0.1256570

2B-GS

C	-0.2971910	3.1330100	0.0000000
C	-0.0000000	0.7395230	0.0000000
C	-1.3785200	0.5149650	0.0000000
C	-2.2448280	1.6608430	0.0000000
C	-1.7369610	2.9073600	0.0000000
H	1.9793390	-0.0277680	0.0000000
C	0.9243700	-0.2918370	0.0000000
C	-1.8087260	-0.8183610	0.0000000
H	-3.3263870	1.5012850	0.0000000
H	-2.3541430	3.8041180	0.0000000
C	-0.9162310	-1.8664100	0.0000000
C	0.4815880	-1.6230640	0.0000000
H	-2.8817020	-1.0255000	0.0000000
H	-1.2954320	-2.8860610	0.0000000
O	0.2497300	4.2113510	0.0000000
O	0.5039450	2.0079080	0.0000000
N	1.3756280	-2.6614740	0.0000000
C	0.9027360	-4.0275040	0.0000000
H	1.7641430	-4.7042540	0.0000000
H	0.2962710	-4.2516680	0.8938270
H	0.2962710	-4.2516680	-0.8938270
C	2.7941280	-2.3835780	0.0000000
H	3.0969030	-1.8112920	0.8932430
H	3.3468420	-3.3293380	0.0000000
H	3.0969030	-1.8112920	-0.8932430

2B-ES1

C	-0.3995520	3.1451720	0.0000000
C	-0.0000000	0.7530100	0.0000000
C	-1.4222500	0.4861680	0.0000000
C	-2.3128040	1.5699500	0.0000000
C	-1.7912560	2.8770580	0.0000000
H	1.9773180	0.0291650	0.0000000
C	0.9283090	-0.2578960	0.0000000
C	-1.8055530	-0.8893190	0.0000000
H	-3.3887990	1.3951860	0.0000000
H	-2.4396170	3.7518710	0.0000000
C	-0.8807420	-1.9000050	0.0000000
C	0.5202470	-1.6127700	0.0000000
H	-2.8710020	-1.1261180	0.0000000
H	-1.2248440	-2.9326120	0.0000000
O	0.1729450	4.2226250	0.0000000
O	0.4772250	2.0137000	0.0000000
N	1.4397590	-2.6203890	0.0000000
C	1.0161260	-4.0049010	0.0000000
H	1.9002820	-4.6503750	0.0000000
H	0.4170920	-4.2438480	0.8944200
H	0.4170920	-4.2438480	-0.8944200
C	2.8531930	-2.3066560	0.0000000
H	3.1345670	-1.7243480	0.8930810
H	3.4293710	-3.2374750	0.0000000
H	3.1345670	-1.7243480	-0.8930810

3B-GS

C	0.0000000	0.6685970	2.4985220
C	0.0000000	0.7125190	-0.0165870
C	-0.0000000	-0.7125190	-0.0165870
C	0.0000000	-1.4425370	1.2406010
C	-0.0000000	-0.6685970	2.4985220
C	0.0000000	1.4078180	-1.2210140
C	-0.0000000	-1.4078180	-1.2210140
H	0.0000000	-1.2582650	3.4170500
C	0.0000000	-0.6826090	-2.4396360
C	0.0000000	0.6826090	-2.4396360
H	0.0000000	-1.2529510	-3.3695680
H	0.0000000	1.2529510	-3.3695680
H	0.0000000	1.2582650	3.4170500
O	0.0000000	-2.6841490	1.2816530
O	0.0000000	2.6841490	1.2816530
C	0.0000000	1.4425370	1.2406010
O	0.0000000	-2.7407440	-1.2903680
H	0.0000000	-3.0860690	-0.3490720
O	0.0000000	2.7407440	-1.2903680
H	0.0000000	3.0860690	-0.3490720

3B–ES1

C	0.0000000	0.6815800	2.4907650
C	0.0000000	0.6918320	-0.0008830
C	-0.0000000	-0.6918320	-0.0008830
C	0.0000000	-1.4381550	1.2689810
C	-0.0000000	-0.6815800	2.4907650
C	0.0000000	1.3999020	-1.2475670
C	-0.0000000	-1.3999020	-1.2475670
H	0.0000000	-1.2575180	3.4171810
C	0.0000000	-0.6904530	-2.4620410
C	0.0000000	0.6904530	-2.4620410
H	0.0000000	-1.2641170	-3.3893090
H	0.0000000	1.2641170	-3.3893090
H	0.0000000	1.2575180	3.4171810
O	0.0000000	-2.6974750	1.2518690
O	0.0000000	2.6974750	1.2518690
C	0.0000000	1.4381550	1.2689810
O	0.0000000	-2.7172900	-1.2589090
H	0.0000000	-3.0155170	-0.2670810
O	0.0000000	2.7172900	-1.2589090
H	0.0000000	3.0155170	-0.2670810

4B-GS

C	0.0853430	2.6077630	0.0000000
C	0.7523730	1.3608970	0.0000000
C	2.1728550	1.2603640	0.0000000
C	-1.2865780	2.6623030	0.0000000
C	0.0000000	0.1672360	0.0000000
C	-1.4139600	0.2534860	0.0000000
C	-2.0425800	1.4743620	0.0000000
C	-2.2237900	-0.9863240	0.0000000
C	-0.1238350	-2.3348380	0.0000000
C	0.6611940	-1.0877630	0.0000000
C	2.0331040	-1.1416940	0.0000000
C	2.7992140	0.0356440	0.0000000
H	3.8903710	-0.0221830	0.0000000
H	2.5148280	-2.1211480	0.0000000
H	-1.7961990	3.6266710	0.0000000
H	-3.1338870	1.4957430	0.0000000
H	-2.0563500	-3.0317520	0.0000000
N	-1.5044500	-2.1720850	0.0000000
O	-3.4437200	-1.0020850	0.0000000
O	0.3629090	-3.4543280	0.0000000
H	0.6830600	3.5197340	0.0000000
O	2.8482090	2.4356220	0.0000000
H	3.8101070	2.2752380	0.0000000

4B-ES1

C	0.3567910	2.5881520	0.0000000
C	0.8821710	1.2939000	0.0000000
C	2.2722200	1.0252020	0.0000000
C	-1.0476530	2.7823260	0.0000000
C	0.0000000	0.1696960	0.0000000
C	-1.3907300	0.3895360	0.0000000
C	-1.9108110	1.7150340	0.0000000
C	-2.3044070	-0.7411570	0.0000000
C	-0.3623450	-2.2929890	0.0000000
C	0.5328500	-1.1311360	0.0000000
C	1.9428270	-1.3649730	0.0000000
C	2.7943530	-0.2976380	0.0000000
H	3.8780830	-0.4414550	0.0000000
H	2.2887780	-2.3987050	0.0000000
H	-1.4385860	3.8009740	0.0000000
H	-2.9938040	1.8383560	0.0000000
H	-2.3464580	-2.7983480	0.0000000
N	-1.7066140	-2.0020630	0.0000000
O	-3.5308940	-0.6426330	0.0000000
O	0.0534620	-3.4518600	0.0000000
H	1.0326390	3.4428110	0.0000000
O	3.0906930	2.0875560	0.0000000
H	4.0279700	1.8105940	0.0000000

5B-GS

C	1.0958690	1.9668960	-0.2795670
C	0.8507870	0.5811710	-0.1229620
C	1.9095060	-0.3917260	-0.1035020
C	0.0664180	2.8758210	-0.2824600
C	-0.4891360	0.1398820	-0.0447520
C	-1.5346110	1.0975290	-0.0346410
C	-1.2628030	2.4390570	-0.1375420
C	-2.9430770	0.6508120	0.0680630
C	-2.1706920	-1.7190800	0.0704390
C	-0.7759710	-1.2494580	-0.0118390
C	0.2479660	-2.1588320	-0.0758040
C	1.5871820	-1.7348890	-0.1209350
H	2.3758870	-2.4858650	-0.1301160
H	0.0002900	-3.2219630	-0.0630490
H	0.2756700	3.9383610	-0.4142780
H	-2.0976030	3.1420040	-0.1297220
H	-4.1058960	-1.0366450	0.1788160
N	-3.1364280	-0.7207550	0.1166750
N	3.2376060	0.0606020	-0.0997020
C	4.2499460	-0.8768990	-0.5394930
H	4.4505020	-1.6751430	0.2035180
H	3.9511860	-1.3404290	-1.4884700
H	5.1873970	-0.3273260	-0.7033520
C	3.6630440	0.7676240	1.1032690
H	4.5458340	1.3837600	0.8781940
H	2.8684900	1.4202240	1.4793890
H	3.9300030	0.0542920	1.9077450
O	-3.8964410	1.4117540	0.1074190
O	-2.5008990	-2.8940440	0.1010510
H	2.1221450	2.3006720	-0.4348960

5B-ES1

C	1.1060810	1.9558390	-0.2993340
C	0.8569210	0.5897440	-0.1183090
C	1.8904210	-0.4112340	-0.0855420
C	0.0436910	2.8869130	-0.2903370
C	-0.4985590	0.1453460	-0.0277840
C	-1.5444530	1.0935740	-0.0268200
C	-1.2583290	2.4731940	-0.1403280
C	-2.9263090	0.6567230	0.0661290
C	-2.1654120	-1.7104960	0.0823360
C	-0.7775640	-1.2373170	-0.0036790
C	0.2517260	-2.2069610	-0.1014150
C	1.5554620	-1.8001020	-0.1565050
H	2.3550070	-2.5399780	-0.1713130
H	-0.0342650	-3.2589120	-0.1037020
H	0.2699680	3.9484360	-0.4053400
H	-2.0937580	3.1733610	-0.1236290
H	-4.0941480	-1.0338180	0.1847230
N	-3.1224940	-0.7252640	0.1236560
N	3.2093070	-0.0515840	0.0128700
C	4.2585230	-0.9372030	-0.4479010
H	4.5434030	-1.6768510	0.3234220
H	3.9483180	-1.4619270	-1.3586130
H	5.1460770	-0.3322400	-0.6809320
C	3.6661780	0.9494740	0.9641330
H	4.2862530	1.7118760	0.4678370
H	2.8162880	1.4347890	1.4514150
H	4.2794530	0.4517930	1.7357890
O	-3.9001330	1.4081510	0.0976230
O	-2.4623480	-2.9046980	0.1175340
H	2.1192930	2.3088330	-0.4844570

6B-GS

C	1.2356270	-0.6488990	0.0003700
C	1.6743780	0.7047560	-0.0059110
C	0.7220400	1.7680540	-0.0267070
C	-0.5876800	1.4282690	-0.0424730
C	-1.0560670	0.0445410	-0.0409940
C	-0.1449010	-0.9886000	-0.0112590
H	1.0479390	2.8081280	-0.0290690
H	-1.3288940	2.2248410	-0.0592450
H	-0.4354980	-2.0351970	0.0035810
N	-2.4112550	-0.1844460	-0.0755300
C	-2.8737620	-1.5511570	-0.0131150
H	-2.5755810	-2.0460970	0.9293950
H	-3.9665930	-1.5704530	-0.0855160
H	-2.4671490	-2.1405630	-0.8506320
C	-3.3635900	0.8942120	0.0944420
H	-3.2997590	1.6307390	-0.7215360
H	-4.3752470	0.4744620	0.0745580
H	-3.2370060	1.4223340	1.0554110
N	2.9860560	0.7301640	0.0123330
N	2.2992260	-1.4234080	0.0229870
O	3.3604160	-0.5664280	0.0298000

6B-ES1

C	-1.2440580	-0.6354820	0.0000050
C	-1.6782460	0.7273100	-0.0000030
C	-0.7210930	1.7674640	-0.0000150
C	0.6433050	1.4640120	-0.0000150
C	1.0746670	0.1151110	-0.0000060
C	0.1211740	-0.9560920	0.0000040
H	-1.0540830	2.8053780	-0.0000250
H	1.3639400	2.2758690	-0.0000280
H	0.4298530	-1.9987830	0.0000190
N	2.3955880	-0.2043890	-0.0000010
C	2.8524550	-1.5869060	-0.0000210
H	2.5049410	-2.1229250	-0.8950710
H	3.9464430	-1.5959430	-0.0000550
H	2.5049950	-2.1229360	0.8950450
C	3.3842760	0.8549490	0.0000200
H	3.2711210	1.4905230	0.8923620
H	4.3879360	0.4199030	0.0000510
H	3.2711730	1.4905140	-0.8923350
N	-2.9956270	0.7570000	0.0000030
N	-2.2845130	-1.4412750	0.0000140
O	-3.3786680	-0.6153930	0.0000130

7B-GS

C	0.9372980	-0.5516140	-0.0097250
C	1.3227400	0.8314530	-0.0132490
C	0.3109500	1.8355420	-0.0364770
C	-0.9894270	1.4512670	-0.0580020
C	-1.3954040	0.0572590	-0.0618190
C	-0.4318230	-0.9284760	-0.0261360
H	0.5961420	2.8880000	-0.0353060
H	-1.7586580	2.2210080	-0.0760260
H	-0.6730490	-1.9880580	-0.0110830
N	-2.7406710	-0.2411110	-0.1081420
C	-3.1338670	-1.6271440	-0.0102450
H	-2.8284310	-2.0823790	0.9507390
H	-4.2230530	-1.7035560	-0.1022920
H	-2.6843240	-2.2172210	-0.8239790
C	-3.7349590	0.7831320	0.1373310
H	-3.7096910	1.5682900	-0.6333830
H	-4.7290460	0.3238490	0.0984950
H	-3.6188910	1.2601780	1.1270640
S	3.3221750	-0.4507220	0.0341630
N	2.6376930	1.0240010	0.0095060
N	1.9831410	-1.3781870	0.0170770

7B-ES1

C	0.9459830	-0.5376720	0.0000010
C	1.3179050	0.8558720	-0.0000010
C	0.2961420	1.8370980	-0.0000020
C	-1.0496220	1.4835700	-0.0000020
C	-1.4186760	0.1182360	0.0000000
C	-0.4057550	-0.8969010	0.0000030
H	0.5872390	2.8883690	-0.0000030
H	-1.8043460	2.2647410	-0.0000040
H	-0.6616530	-1.9541850	0.0000080
N	-2.7219010	-0.2655100	0.0000000
C	-3.1061190	-1.6688830	-0.0000060
H	-2.7304540	-2.1871390	0.8945390
H	-4.1982570	-1.7353650	-0.0000150
H	-2.7304390	-2.1871350	-0.8945460
C	-3.7615810	0.7437070	0.0000050
H	-3.6824870	1.3843830	-0.8925180
H	-4.7423290	0.2588430	0.0000120
H	-3.6824760	1.3843850	0.8925250
S	3.3489100	-0.4720980	0.0000000
N	2.6334130	1.0622740	-0.0000020
N	1.9674840	-1.3929770	0.0000020

8B-GS

C	0.7248270	0.5126940	0.0000000
C	1.1583680	-0.8071480	0.0000000
C	0.2341940	-1.8364470	0.0000000
C	-1.1162550	-1.5146760	0.0000000
C	-1.5657790	-0.1714130	0.0000000
C	-0.6024670	0.8658950	-0.0000010
C	1.9296520	1.3993320	0.0000000
C	2.6384280	-0.8201010	0.0000000
H	0.5586440	-2.8786030	0.0000000
H	-1.8435290	-2.3240920	0.0000000
H	-0.8807670	1.9182730	0.0000000
O	3.4097670	-1.7559090	0.0000000
O	1.9841900	2.6108180	0.0000000
N	3.0158340	0.5313830	0.0000000
H	3.9849040	0.8410790	0.0000000
N	-2.9038660	0.1211150	0.0000000
C	-3.3379400	1.5012040	0.0000000
H	-2.9819220	2.0411340	-0.8934070
H	-4.4327810	1.5330320	0.0000000
H	-2.9819220	2.0411340	0.8934070
C	-3.8797140	-0.9454540	0.0000000
H	-3.7862960	-1.5853220	0.8938220
H	-4.8853440	-0.5113780	0.0000000
H	-3.7862970	-1.5853220	-0.8938220

8B-ES1

C	-0.7380190	0.5888520	-0.0000010
C	-1.1584270	-0.7897950	0.0000000
C	-0.2284820	-1.8358130	-0.0000010
C	1.1068720	-1.5306450	-0.0000020
C	1.5475830	-0.1429230	-0.0000020
C	0.6120570	0.9087580	-0.0000010
C	-1.9065570	1.4189340	0.0000020
C	-2.6175120	-0.8474270	0.0000020
H	-0.5797580	-2.8690540	-0.0000010
H	1.8434960	-2.3303550	-0.0000020
H	0.9112730	1.9545180	-0.0000010
O	-3.3719650	-1.8226920	0.0000010
O	-2.0411570	2.6479060	0.0000000
N	-2.9853160	0.4917940	0.0000010
H	-3.9587050	0.7952690	0.0000000
N	2.8805710	0.1109130	-0.0000010
C	3.3463000	1.4856120	0.0000000
H	2.9834460	2.0186580	0.8924700
H	4.4399970	1.5005330	0.0000000
H	2.9834470	2.0186590	-0.8924700
C	3.8690010	-0.9581980	0.0000010
H	3.7748120	-1.5897420	-0.8952540
H	4.8684770	-0.5135370	0.0000020
H	3.7748100	-1.5897410	0.8952560

9B-GS

C	2.5018490	1.0113390	0.0339700
N	3.1641600	-0.1659660	-0.0443350
C	4.5097330	-0.2150050	-0.1631620
C	5.2693580	0.9230030	-0.2001580
C	4.6132270	2.1618460	-0.1066270
C	3.2463670	2.2069680	0.0085390
B	2.3680020	-1.5188360	0.0984540
F	2.8706650	-2.4227440	-0.8136820
N	1.1463430	1.0687050	0.1197130
C	0.4482420	-0.0300000	-0.0182220
O	0.9649750	-1.2245280	-0.1855560
F	2.5205730	-1.9578070	1.4032310
H	4.9276410	-1.2198210	-0.2270770
H	6.3519670	0.8547680	-0.2953960
H	5.1896270	3.0886060	-0.1286370
H	2.6916690	3.1414780	0.0765590
C	-1.0123030	0.0264470	-0.0245090
C	-1.6808290	1.2489190	0.0765770
C	-3.0600190	1.3092350	0.0758800
C	-3.8338670	0.1324120	-0.0287320
C	-3.1509560	-1.0983860	-0.1297160
C	-1.7702150	-1.1423150	-0.1266250
H	-1.0898860	2.1618160	0.1576450
H	-3.5449450	2.2802630	0.1570460
N	-5.2033510	0.1842120	-0.0312390
H	-3.7063900	-2.0309400	-0.2086130
H	-1.2555960	-2.1008080	-0.2021000
C	-5.9715600	-1.0364260	-0.1291790
C	-5.8784000	1.4564110	0.0901560
H	-6.9613430	1.2918800	0.0705810
H	-5.6283240	1.9641620	1.0373060
H	-5.6231370	2.1350070	-0.7412320
H	-7.0392000	-0.7909340	-0.1195310
H	-5.7563790	-1.5793790	-1.0648320
H	-5.7704700	-1.7160760	0.7163890

9B–ES1

C	2.4768430	1.0218180	-0.0061590
N	3.1571810	-0.1880940	-0.0730460
C	4.4985860	-0.2054460	-0.1614450
C	5.2535120	0.9456520	-0.1756350
C	4.5966720	2.1947770	-0.0768270
C	3.2256630	2.2227750	0.0081020
B	2.3574070	-1.5218030	0.1240830
F	2.9351410	-2.5089680	-0.6528600
N	1.1423640	1.0590640	0.0249210
C	0.4359620	-0.0819610	-0.1015480
O	0.9801380	-1.2780200	-0.2994300
F	2.3977330	-1.8468050	1.4788640
H	4.9413550	-1.2008990	-0.2219290
H	6.3378720	0.8774680	-0.2545750
H	5.1704430	3.1224070	-0.0707960
H	2.6626000	3.1536480	0.0749170
C	-0.9859110	-0.0029620	-0.0777490
C	-1.6563010	1.2459910	0.0644200
C	-3.0232670	1.3141410	0.0892580
C	-3.8165700	0.1375640	-0.0265880
C	-3.1451040	-1.1122680	-0.1713610
C	-1.7785270	-1.1782600	-0.1960430
H	-1.0470880	2.1449180	0.1545110
H	-3.5017470	2.2855170	0.2002940
N	-5.1772570	0.2013540	-0.0007260
H	-3.7200230	-2.0321220	-0.2618530
H	-1.2666030	-2.1347100	-0.3029470
C	-5.9697140	-1.0061350	-0.1150980
C	-5.8447210	1.4775750	0.1511570
H	-6.9280180	1.3197580	0.1483030
H	-5.5696960	1.9650150	1.1012240
H	-5.5942130	2.1631530	-0.6751930
H	-7.0317780	-0.7438240	-0.0743050
H	-5.7795310	-1.5233880	-1.0698170
H	-5.7563420	-1.7076540	0.7082080

10B-GS

N	4.2012520	-0.0587360	-0.0455890
C	5.5343830	0.1431970	-0.1452090
C	6.0707700	1.4019410	-0.1707150
C	5.1951650	2.4977480	-0.0859210
C	3.8424080	2.2887010	0.0095870
C	3.3312810	0.9754670	0.0238200
H	6.1321640	-0.7665780	-0.2032600
H	7.1485120	1.5355070	-0.2505790
H	5.5902480	3.5152750	-0.0992590
H	3.1234880	3.1042580	0.0698040
N	1.9885970	0.7807380	0.0893100
B	3.6749870	-1.5394560	0.0847470
C	1.5100960	-0.4310970	-0.0441390
O	2.2371760	-1.5144420	-0.1820280
F	3.9228190	-1.9575130	1.3814580
F	4.3304830	-2.3199200	-0.8442620
C	0.0748240	-0.6721910	-0.0793590
C	-0.8163860	0.3275260	0.0099710
C	-2.2683590	0.2175000	-0.0036840
C	-3.0457900	1.3755600	0.0883860
C	-4.4291630	1.3362080	0.0800390
C	-5.1095630	0.1078330	-0.0255560
C	-4.3287150	-1.0652290	-0.1141030
C	-2.9494900	-1.0020560	-0.1037110
H	-2.5450070	2.3437230	0.1711240
H	-4.9840490	2.2695090	0.1566240
N	-6.4810380	0.0503630	-0.0407810
H	-4.8089720	-2.0390830	-0.1911600
H	-2.3854440	-1.9341060	-0.1732960
C	-7.1489790	-1.2297860	-0.0958410
C	-7.2516470	1.2635240	0.1041830
H	-8.3188810	1.0188310	0.0656470
H	-7.0508850	1.7679420	1.0657150
H	-7.0397000	1.9782090	-0.7085980
H	-8.2327820	-1.0694260	-0.1015940
H	-6.8859190	-1.7862230	-1.0108120
H	-6.9020910	-1.8628510	0.7743510
H	-0.4087230	1.3391120	0.1046630
H	-0.2107000	-1.7200290	-0.1787020

10B-ES1

N	4.1817880	-0.0833870	-0.0623040
C	5.5095450	0.1230720	-0.1437140
C	6.0529610	1.3830760	-0.1725000
C	5.1801500	2.4890400	-0.0991360
C	3.8276840	2.2846430	-0.0194170
C	3.3008450	0.9678700	-0.0153500
H	6.1110230	-0.7859510	-0.1845420
H	7.1324320	1.5099320	-0.2416310
H	5.5807490	3.5045130	-0.1081320
H	3.1119190	3.1045230	0.0306270
N	1.9774150	0.7765780	0.0220440
B	3.6260990	-1.5466360	0.1226950
C	1.4811040	-0.4664400	-0.1227220
O	2.2377100	-1.5444160	-0.3081680
F	3.7295930	-1.8567580	1.4766270
F	4.3814380	-2.4051970	-0.6547410
C	0.0846750	-0.6459710	-0.1384620
C	-0.8156850	0.4162020	-0.0043470
C	-2.2196800	0.2836860	-0.0103920
C	-3.0492150	1.4353310	0.1321830
C	-4.4138410	1.3579610	0.1358520
C	-5.0722560	0.1011530	-0.0056280
C	-4.2624960	-1.0558210	-0.1497520
C	-2.8941070	-0.9678040	-0.1522920
H	-2.5654010	2.4085380	0.2427480
H	-4.9966950	2.2702810	0.2489630
N	-6.4308960	0.0174410	-0.0019050
H	-4.7258980	-2.0346530	-0.2600400
H	-2.3093090	-1.8809440	-0.2641610
C	-7.0807330	-1.2692060	-0.1454110
C	-7.2378660	1.2114150	0.1480740
H	-8.2966470	0.9337860	0.1260320
H	-7.0362160	1.7174120	1.1065510
H	-7.0545550	1.9280300	-0.6693580
H	-8.1662120	-1.1293160	-0.1181540
H	-6.8194730	-1.7473010	-1.1039240
H	-6.8015000	-1.9545350	0.6718850
H	-0.3850690	1.4126800	0.1145170
H	-0.2552660	-1.6745670	-0.2632420

11B-GS

C	0.0000000	0.4345900	0.0000000
C	-0.0583170	-0.9993340	0.0000000
H	0.8917050	-1.5431820	0.0000000
C	-1.1600590	-1.7755090	0.0000000
N	-2.5029090	-1.3447680	0.0000000
C	-3.2073410	-2.4232400	0.0000000
C	-1.1142800	-3.2533980	0.0000000
N	-2.4699090	-3.5908850	0.0000000
O	-0.1772490	-4.0337910	0.0000000
H	-4.2973830	-2.4474880	0.0000000
H	-2.8202490	-4.5440350	0.0000000
C	-1.0591410	1.3178820	0.0000000
H	-2.1214280	1.0900230	0.0000000
N	-0.5877090	2.5968550	0.0000000
H	-1.1671400	3.4295580	0.0000000
C	0.7966780	2.5861560	0.0000000
C	1.2020470	1.2402160	0.0000000
C	1.7048500	3.6449360	0.0000000
C	2.5705810	0.9442030	0.0000000
C	3.0502670	3.3258510	0.0000000
H	1.3690440	4.6839890	0.0000000
C	3.4795780	1.9859330	0.0000000
H	2.9167560	-0.0916500	0.0000000
H	3.7921420	4.1262330	0.0000000
H	4.5490540	1.7687400	0.0000000

11B-ES1

C	0.0000000	0.3707740	0.0000000
C	0.2578090	-1.0017160	0.0000000
H	1.2838750	-1.3741700	0.0000000
C	-0.7597790	-1.9860670	0.0000000
N	-2.1137880	-1.7905100	0.0000000
C	-2.6618770	-2.9958070	0.0000000
C	-0.4584550	-3.4154340	0.0000000
N	-1.7307560	-3.9948410	0.0000000
O	0.6226740	-4.0107060	0.0000000
H	-3.7322780	-3.1896430	0.0000000
H	-1.8973440	-4.9971060	0.0000000
C	-1.2781420	0.9901440	0.0000000
H	-2.2464320	0.4947070	0.0000000
N	-1.1199670	2.3324960	0.0000000
H	-1.8838120	3.0027320	0.0000000
C	0.2322700	2.6672020	0.0000000
C	0.9602240	1.4643780	0.0000000
C	0.8444790	3.9121540	0.0000000
C	2.3509120	1.5096040	0.0000000
C	2.2351730	3.9343990	0.0000000
H	0.2652070	4.8376120	0.0000000
C	2.9807790	2.7497730	0.0000000
H	2.9368890	0.5882740	0.0000000
H	2.7531600	4.8949420	0.0000000
H	4.0705680	2.8018690	0.0000000

12B-GS

C	-0.5352030	3.2501330	0.0000000
C	-1.8919700	2.9380340	0.0000000
C	-2.3081420	1.6073440	0.0000000
C	-1.3714330	0.5870180	0.0000000
C	0.0000000	0.8783100	0.0000000
C	0.3930040	2.2246250	0.0000000
H	-0.2325680	4.2977750	0.0000000
H	-3.3757160	1.3698250	0.0000000
H	-1.6904440	-0.4551470	0.0000000
H	1.4585110	2.4667760	0.0000000
C	1.0335170	-0.1437830	0.0000000
H	2.0686710	0.2145340	0.0000000
C	0.9162740	-1.4833790	0.0000000
N	-0.2572140	-2.2668210	0.0000000
C	0.1465260	-3.4889700	0.0000000
C	2.1023810	-2.3780280	0.0000000
N	1.5192820	-3.6449210	0.0000000
O	3.2948230	-2.1304110	0.0000000
O	-2.7713520	3.9764230	0.0000000
H	-0.5161110	-4.3548760	0.0000000
H	2.0429820	-4.5150640	0.0000000
H	-3.6872910	3.6424520	0.0000000

12B-ES1

C	-3.0985310	1.1528350	0.1019470
C	-3.4612410	-0.1975280	-0.0456450
C	-2.4824840	-1.1874790	-0.1543700
C	-1.1451060	-0.8490820	-0.1144770
C	-0.7414990	0.5087130	0.0601780
C	-1.7725610	1.4936590	0.1453660
H	-3.8883260	1.9012270	0.1784730
H	-2.7793660	-2.2324380	-0.2836080
H	-0.3734910	-1.6143930	-0.2003290
H	-1.4827920	2.5399470	0.2662200
C	0.6058060	0.9013500	0.1527330
H	0.8364700	1.9681300	0.2229960
C	1.7370310	0.0552060	0.0757900
N	1.8036410	-1.2916540	0.2643060
C	3.0782430	-1.6393420	0.1333390
C	3.0840970	0.5874500	-0.1312110
N	3.8854680	-0.5670350	-0.0598340
O	3.4912060	1.7427050	-0.2562050
O	-4.7866780	-0.4734550	-0.0855060
H	3.4426230	-2.6643730	0.1438520
H	4.8948200	-0.5624420	-0.1831190
H	-4.9324480	-1.4335380	-0.1840000

13B-GS

C	2.7815550	-4.2445780	0.0000000
C	1.4824210	-3.7571860	0.0000000
C	3.8517220	-3.3559580	0.0000000
C	3.6159040	-1.9829060	0.0000000
C	1.2321670	-2.3789190	0.0000000
C	2.3186830	-1.4920890	0.0000000
C	-0.1564190	-1.9418200	0.0000000
C	-0.6728700	-0.6990840	0.0000000
N	0.0000000	0.5329670	0.0000000
C	-0.9338800	1.4288580	0.0000000
C	-2.1458580	-0.4547910	0.0000000
N	-2.2182560	0.9352390	0.0000000
H	4.8757960	-3.7342480	0.0000000
H	-3.0644080	1.5015260	0.0000000
O	-3.0752510	-1.2376770	0.0000000
H	2.9599350	-5.3211920	0.0000000
H	0.6386550	-4.4514030	0.0000000
H	4.4561210	-1.2861270	0.0000000
H	2.1284010	-0.4194920	0.0000000
H	-0.9122990	-2.7347900	0.0000000
C	-0.6685580	2.8617790	0.0000000
H	0.3953450	3.1532200	0.0000000
N	-1.6390750	3.6893270	0.0000000
C	-1.2550280	5.0543980	0.0000000
H	-0.1536240	5.2396440	0.0000000
O	-2.0524540	5.9531660	0.0000000

13B-ES1

C	3.1359330	-4.0095410	0.0000000
C	1.8046030	-3.6596790	0.0000000
C	4.1188760	-3.0113050	0.0000000
C	3.7561420	-1.6644040	0.0000000
C	1.4095060	-2.2939410	0.0000000
C	2.4233550	-1.2968080	0.0000000
C	0.0302500	-1.9866910	0.0000000
C	-0.5654170	-0.7181530	0.0000000
N	0.0000000	0.4990670	0.0000000
C	-1.0199840	1.3913810	0.0000000
C	-2.0513940	-0.5924510	0.0000000
N	-2.2407140	0.7793090	0.0000000
H	5.1744910	-3.2905400	0.0000000
H	-3.1273200	1.2862170	0.0000000
O	-2.9016410	-1.4668520	0.0000000
H	3.4261120	-5.0614260	0.0000000
H	1.0295350	-4.4295530	0.0000000
H	4.5290410	-0.8938110	0.0000000
H	2.1252990	-0.2485080	0.0000000
H	-0.6857760	-2.8144480	0.0000000
C	-0.8998060	2.8050850	0.0000000
H	0.1197560	3.2224650	0.0000000
N	-1.9789300	3.5345680	0.0000000
C	-1.7728650	4.9096550	0.0000000
H	-0.7015470	5.2335450	0.0000000
O	-2.6692690	5.7251740	0.0000000

References

(S1) Chołuj, M.; Alam, M. M.; Beerepoot, M. T. P.; Sitkiewicz, S. P.; Matito, E.; Ruud, K.; Zaleśny, R. Choosing Bad Versus Worse: Predictions of Two-Photon-Absorption Strengths Based on Popular Density Functional Approximations. *J. Chem. Theory Comput.* **2022**, *18*, 1046–1060.

(S2) Nizomov, N.; Kholov, A. U.; Ishchenko, A. A.; Ishchenko, V. V.; Khilya, V. P. Electronic Structure and Spectral Fluorescence Properties of Umbelliferone and Herniarin. *J. Appl. Spectrosc.* **2007**, *74*, 626–634.

(S3) Husain, M. M.; Sindhu, R.; Tandon, H. C. Photophysical Properties and Estimation of Ground and Excited State Dipole Moments of 7-diethylamino and 7-diethylamino-4-methyl Coumarin Dyes From Absorption and Emission Spectra. *Eur. J. Chem.* **2012**, *3*, 87–93.

(S4) Singh, I.; Ogata, R.; Moore, R.; Chang, C.; Scheuer, P. Electronic Spectra of Substituted Naphthoquinones. *Tetrahedron* **1968**, *24*, 6053–6073.

(S5) Uchiyama, S.; Takehira, K.; Kohtani, S.; Santa, T.; Nakagaki, R.; Tobita, S.; Imai, K. Photophysical Study of 5-substituted Benzofurazan Compounds as Fluorogenic Probes. *Phys. Chem. Chem. Phys.* **2002**, *4*, 4514–4522.

(S6) Ferraro, V.; Girotto, M.; Bortoluzzi, M. N,N-dimethyl-4-amino-2,1,3-benzothiadiazole: Synthesis and Luminescent Solvatochromism. *Chem. Proc.* **2022**, *8*.

(S7) Qu, Z.; Li, P.; Zhang, X.; Wang, E.; Wang, Y.; Zhou, P. Excited-state Proton Transfer of 4-hydroxyl-1, 8-naphthalimide Derivatives: A Combined Experimental and Theoretical Investigation. *J. Lumin.* **2016**, *177*, 197–203.

(S8) Kucheryavy, P.; Li, G.; Vyas, S.; Hadad, C.; Glusac, K. D. Electronic Properties of 4-substituted Naphthalimides. *J. Phys. Chem. A* **2009**, *113*, 6453–6461.

(S9) Bucsiová, L.; Hrdlovič, P. Medium Effect of Polymer Matrices on Spectral Properties of 4-aminophthalimide and 4-dimethylaminophthalimide. *J. Macromol. Sci. Chem. A.* **2007**, *44*, 1047–1053.

(S10) Bednarska, J.; Zaleśny, R.; Wielgus, M.; Jędrzejewska, B.; Puttreddy, R.; Rissanen, K.; Bartkowiak, W.; Ågren, H.; Ośmiałowski, B. Two-Photon Absorption of BF2-Carrying Compounds: Insights From Theory and Experiment. *Phys. Chem. Chem. Phys.* **2017**, *19*, 5705–5708.

(S11) Drobizhev, M.; Makarov, N. S.; Tillo, S. E.; Hughes, T. E.; Rebane, A. Two-Photon Absorption Properties of Fluorescent Proteins. *Nat. Methods* **2011**, *8*, 393–399.

(S12) Ai, H.-w.; Shaner, N. C.; Cheng, Z.; Tsien, R. Y.; Campbell, R. E. Exploration of New Chromophore Structures Leads to the Identification of Improved Blue Fluorescent Proteins. *Biochemistry* **2007**, *46*, 5904–5910.
